# KiMoPack: A python Package for Kinetic Modeling of the Chemical Mechanism

**DOI:** 10.1021/acs.jpca.2c00907

**Published:** 2022-06-14

**Authors:** Carolin Müller, Torbjörn Pascher, Axl Eriksson, Pavel Chabera, Jens Uhlig

**Affiliations:** † Institute for Physical Chemistry, Friedrich Schiller University Jena, Helmholtzweg 4, 07743 Jena, Germany; ‡ Leibniz Institute of Photonic Technology (IPHT) Jena, Albert-Einstein-Strasse 9, 07745 Jena, Germany; ¶ Department of Chemical Physics, Lund University, SE-22100 Lund, Sweden

## Abstract

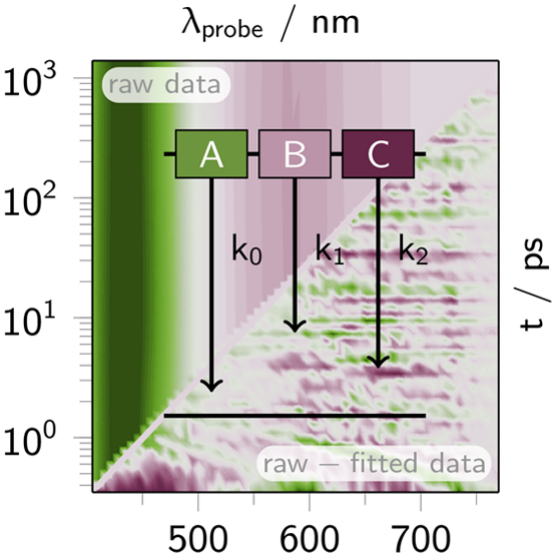

Herein, we present KiMoPack, an analysis tool for the **ki**netic **mo**deling of transient spectroscopic data. KiMoPack enables a state-of-the-art analysis routine including data preprocessing and standard fitting (global analysis), as well as fitting of complex (target) kinetic models, interactive viewing of (fit) results, and multiexperiment analysis via user accessible functions and a graphical user interface (GUI) enhanced interface. To facilitate its use, this paper guides the user through typical operations covering a wide range of analysis tasks, establishes a typical workflow and is bridging the gap between ease of use for less experienced users and introducing the advanced interfaces for experienced users. KiMoPack is open source and provides a comprehensive front-end for preprocessing, fitting and plotting of 2-dimensional data that simplifies the access to a powerful python-based data-processing system and forms the foundation for a well documented, reliable, and reproducible data analysis.

## Introduction

In many chemical disciplines, kinetic modeling is used to extract and understand the mechanisms underlying time-resolved spectroscopic data. It is extensively used in the analysis of time-resolved spectra from optical spectroscopy,^1−3^ but also in, e.g., X-ray spectroscopy,^4,5^ spectroelectrochemistry,^6−10^ and photocatalysis.^8,11,12^ Common to all techniques is that a spectrum is distorted by one, or multiple processes. The evolution of the distortion can be used to, e.g., disentangle processes or/and to extract additional information from the system. In the study of photoinduced dynamics including energy and electron transfer, transient/time-resolved spectroscopy can be an essential tool.^1^ One form of time-resolved spectroscopy is to follow the temporal evolution after a pulsed excitation by monitoring changes in optical properties such as absorption, emission or scattering of light. The challenge in working with these inherently multivariate data sets is the need to extract the information in a controlled and reproducible way, not using the proverbial *black box* approach.

In this paper, we present KiMoPack (**ki**netic **mo**deling **pack**age), a software package designed to model photoinduced chemical kinetic measurements, which is also capable to model modulated data obtained by other initiation methods. We have used KiMoPack successfully to analyze datasets obtained from transient X-ray spectroscopy,^5^ photocatalysis,^12^ and spectroelectrochemistry.^6,7,13^ However, in the following discussions, we will focus on transient optical spectroscopy and the associated data handling. The essential design criteria for KiMoPack are to provide a reproducible workflow tool in which preprocessing, model-free analysis, and fast and advanced modeling are combined with a powerful set of plotting and comparison methods. The data work up can be divided into five general stages:

(A) **Preprocessing.** Perform background subtraction, arrival time correction, combine and filter multiple measurements, define limits, suppress scattered light, and visually inspect/compare data.
(B) **Kinetic Model-Free Analysis.** Employ model free analysis methods such as, e.g., singular value decomposition (SVD)^14−19^ to gain insights into the number of processes/states (e.g., chemical species) contributing to the data set.
(C) **Global Analysis.** Express the dynamics using independent first-order exponential decays with guessed parameters and use global analysis^2,20−22^ to optimize the parameters and decompose the time-resolved spectra into kinetic traces and transient spectra, assuming a bi-linearity of the data.
(D) **Target Analysis.** Express different chemical mechanisms through parameter dependent temporally changing concentrations. Often this is achieved through numerically integrating differential rate equations. Optimize the used set of parameter through global analysis and extract the species associated spectra (SAS).^2,20−22^ In KiMoPack, all fitting steps may include external spectra (e.g., spectro-electroctochemical data) and external kinetic information (e.g., measured laser pulse profiles) and can be simultaneously performed on multiple data sets. A confidence interval of the variable parameter set can then be evaluated using an *F*-test, comparing the parameter induced variations (under reoptimization) to the statistical variations.
(E) **Visualization of results.** In KiMoPack, powerful visualization routines are provided to facilitate the refinement and comparison of the results from different kinetic models, create informative report files or publication ready plots. Flexible reporting, data extraction, and postprocessing capabilities simplify interaction with other software packages, e.g., as input in other modeling tools or specialized scientific plotting software.

KiMoPack is an open source python package that uses customizable *Jupyter* notebooks as workflow tools to provide a powerful, but still user-friendly interface for a well documented, flexible, and reproducible analysis of transient data. Its modularity also allows the combination with other tools like the recently introduced DeepSKAN,^23^ which suggests probable kinetic models from time-resolved spectra by artificial intelligence algorithms but does not perform the target analysis. However, we have found that at the current state of development, human intuition and experience often leads faster to the right conclusions. To the best of our knowledge, there is not yet such a comprehensive open source program available for analyzing data in more complicated models than the standard parallel and sequential approaches that simultaneously integrate a comprehensive data pre- and postprocessing and statistically robust error estimation.^24−27^

The program that comes closest to the desired functionality is Glotaran,^24,28,29^ which however is lacking in some flexibility of model building and fitting control, and has significant limitations due to primarily relying on a graphical framework.^24^ In contrast, the herein presented KiMoPack is designed as a very flexible python framework that triggers the analysis tasks through flexible and user-friendly functions, acting as a frontend to the programming language. The source code of KiMoPack is available on Github^30^ and as frozen releases on zenodo.^31^ Typically it is installed through one of the two common package managers conda(32) or pypi.^33^ The code is documented comprehensively on ReadtheDocs.^34^ A few introductory movies are available on the KiMoPack tutorial youtube channel.^35^ The workflow tools and a series of working-along tutorials are available on the Github page.^30^

The aim of this contribution is to present the general workflow and advanced features of KiMoPack that outperform state-of-the-art open-source and commercial programs.^24,36^ This contribution also provides *Jupyter* notebooks that guide through several typical analysis tasks (workflow tools) making KiMoPack easier accessible to users with less programming background while offering the full flexibility of python for more experienced user. Both the tutorials and the following sections focus on the analysis and discussion of transient absorption (TA) data.

## Results

**Figure 1:**
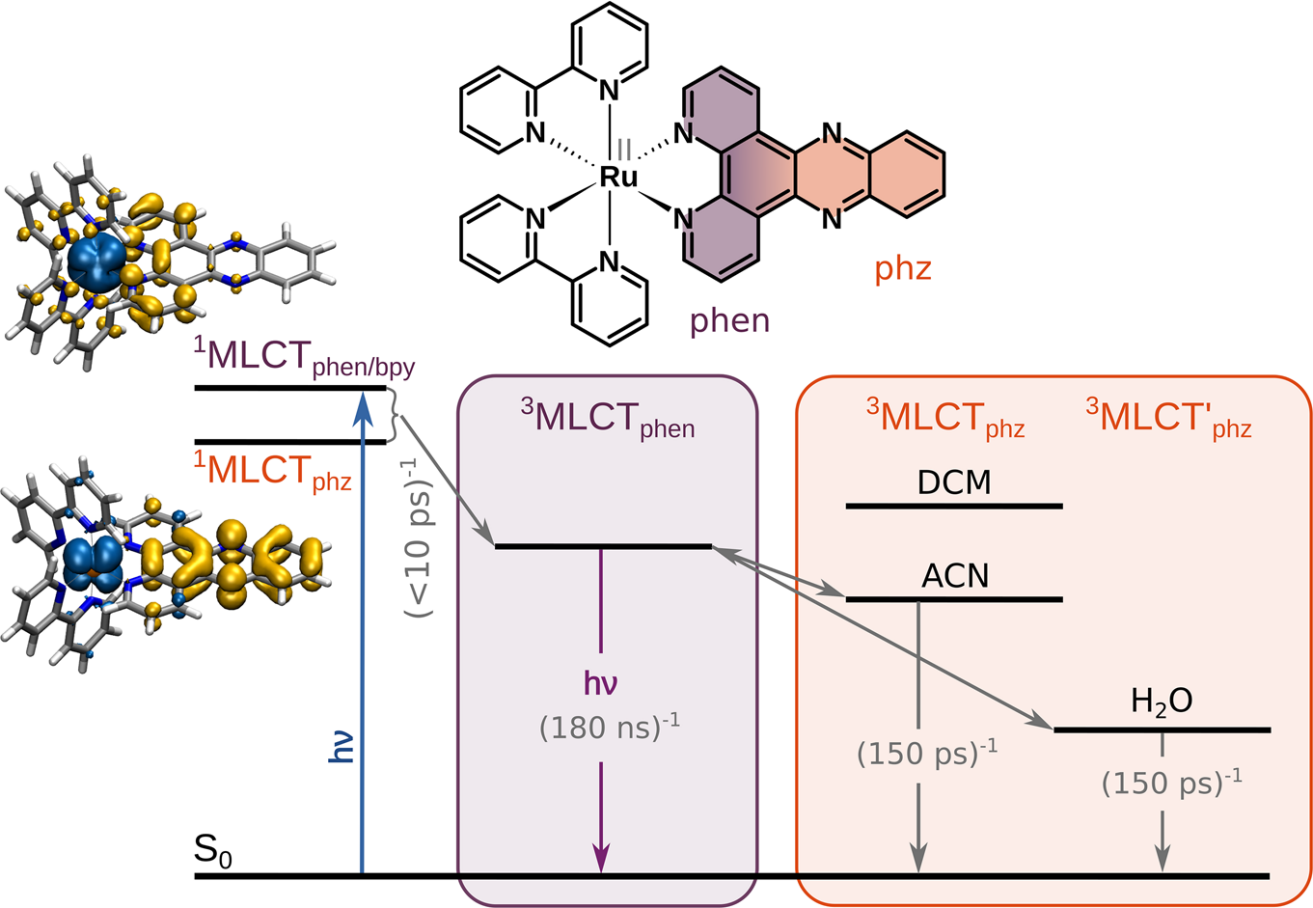
Schematic Jablonski diagrams for the photoinduced relaxation schemes of Ru-dppz in dichloromethane (DCM), acetonitrile (ACN), and water (H_2_O) upon MLCT excitation between 390 and 450 nm.

We will demonstrate the application of KiMoPack for analyzing typical TA data sets using the widely studied ruthenium complex [(tbbpy)_2_Ru(dppz)]^2+^ (Ru-dppz, tbbpy = 4,4′-di-*tert*-butyl-2,2′-bipyridine, and dppz = dipyrido[3.2-a:2′,3′-c]phenazine) as model substance.^37−41^ Ru-dppz is interesting due to its so-called *light-switch* effect in which the relative population of a bright (emissive) and a dark (non-emissive) ^3^MLCT excited state is governed by the local solvent environment (see Figure 1).^38−40,42−46^ The general relaxation models in various solvents are well-known from literature.^42,43,45,47−49^ However, the analysis is challenging in the sense that it requires the handling of multiple data sets, different kinetic models, and the comparison functions. This makes Ru-dppz a suitable reference system to demonstrate the workflow in KiMoPack. The respective data sets are available as Supporting Information (e.g., TA_Ru-dppz_400 nm_ACN.SIA) as are a series of tutorials that allow the reader to follow the analysis of TA data of Ru-dppz step-by-step. The sample data shown here was collected upon 400 nm excitation in dichloromethane (DCM), acetonitrile (ACN) and water (H_2_O) in various levels of complexity.

**Figure 2:**
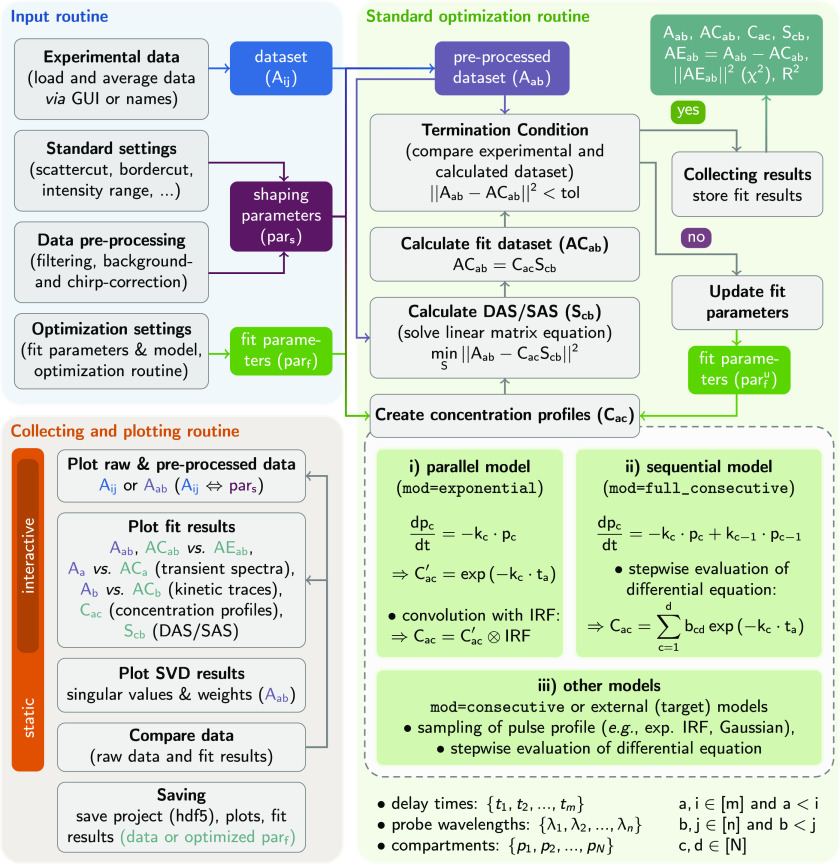
Schematic representation of the processing structure and the three main workflow routines, i.e., the input (blue box) and standard optimization (green box), as well as collecting and plotting routines (orange box) KiMoPack. Individual operations (functions) and their returns are indicated by bright gray and colored boxes, respectively.

The provided *Jupyter* notebooks contain the functions and parameters that are employed and adjusted during a representative analysis session. This allows early users to easily change the values or extend the comments and the experienced users to have a customizable, fast, and reproducible workflow. Typically, a new *Jupyter* notebook is created from the templates/previous analysis for each new analysis session, which then documents the procedures, parameters, and results in a visible way. This procedure also enables very fast and repeated data processing as the entire notebook can be run repeatedly. In the tutorials, several of these analysis sessions are extensively documented, and their individual functionality is explained. An even more comprehensive documentation of all functions and their parameters can be found on ReadtheDocs.^34^ In the following, we will guide the reader through the key analysis steps: reading, preprocessing, kinetic modeling, plotting, and saving of data, for the example of TA data from Ru-dppz. These analysis steps can be divided into three working routines: the input, optimization, and collecting routine of KiMoPack (see Figure 2). This modular structure allows not only flexible workflows but also the concept that future extensions can be made without significant changes to the program core and prior analysis notebooks can be reused and extended.

## Input Routine

In the input routine, the experimental data are loaded and converted into the form of a matrix *A*_*ij*_ (, *m* = number of times, *n* = number of wavelengths). Subsequently, these data can be preprocessed, e.g., chirp-corrected, and a range of parameter is defined that defines, e.g., a spectral or temporal range of interest or certain masks for the subsequent routines of analysis and plotting.

### Import of Experimental Data

KiMoPack supports the work with various formats of time-resolved spectroscopic data. The internal format is based on a pandas DataFrame(s)(50) that assembles *n* probe wavelengths {λ_1_, λ_2_, ..., λ_*n*_} and at *m* times relative to the instant of excitation {*t*_1_, *t*_2_, ..., *t*_*m*_} into a matrix *A*_*ij*_ with the times and wavelength as index. Noteworthy, the import functions support many file formats; i.e., the measured data can be provided transposed, with separate files for temporal and wavelength (or energy) information or can be a single wavelength measured kinetics to name just a few. The file(s) to be processed are selected either via a GUI (pf.TA(’gui’) or pf.TA(’recent’) for a single file or pf.GUI_open() for multiple files) or the filename (e.g., pf.TA(’filename.SIA’)) with a wide range of further importing options. For further details see the *Opening of data* section in the documentation.^51^

#### Single Scan Handling

Often spectroscopic data are collected over multiple separate measurements (scans). In this case a separate inspection and selection of the scans prior to the analysis is often beneficial. The Summarize_scans function displays the sum of the measured intensities in one or two user definable time-spectral windows for each scan and allows an interactive selection of scans with damaged/changed samples regions or scans with particularly strong disturbances. The tutorial 04_KiMoPack_ScanHandling.ipynb is a guide through this procedure. In the tutorial, one of the windows represents the excited state absorption (ESA) and the second the ground state bleach (GSB). In this example, fluctuations in the GSB indicate fluctuations of the laser pump intensity, while changes in the ESA indicate sample degradation, which can be selected and excluded from the average.

### Data Preprocessing

Preprocessing of data typically includes a variety of tasks, including the reading (of many formats), binning, baseline subtraction, filtering, and arrival-time (chirp) correction as well as the suppression of one or multiple (temporal or spectral) regions containing scattered excitation light or artifacts.^52,53^ One important concept of KiMoPack is to apply the shaping during each action, such as plotting or fitting anew to a fresh copy of the unaltered data set. This requires that all necessary parameters are passed to each function. For the user this is simplified by packaging the data, functions, and parameters into a TA object that contains all the parameters. A detailed description of the functions and their arguments can be found in the documentation in the *Shaping of data* section.^54^ Typically, the initial task of inspecting the measured data consists (all optional) of: (i) Importing of a file (creating the TA object), (ii) filtering of obvious spikes (Filter_data-function), (iii) baseline subtraction (Background function), (iv) arrival time correction (time-zero of each probe wavelength, Cor_Chirp function), and (v) plotting of the so processed data (Plot_RAW or Plot_Interactive function). Of these, all but the import are optional and triggered by calling the respective functions.

#### Arrival-Time (Chirp) Correction

An essential preprocessing step is the correction of the temporal axis for different wavelength, often called arrival time or chirp correction. This is initially accomplished by selecting a series of points via a GUI in the 2D-map of the TA data in a selected time-window (Cor_Chirp). Those points are then approximated with a 4^th^ order polynomial and the original data (stored in ds_ori) are interpolated and corrected along this so defined dispersion curve and stored as copy (in the variable ds). This selection can be refined manually at any time or automatically during the fitting procedure. Moreover, correction curves from prior or similar measurements can easily be employed.

## Optimization Routines

In physics, chemistry, and biology, much effort and research are devoted to understanding the dynamics of various processes. To unravel and characterize the processes that contribute to changes in system parameters (e.g., absorption or vibration properties) with time, it is necessary to analyze the respective time-resolved data within physically meaningful models. Modeling of the kinetic development of spectral species is one of the main tasks of KiMoPack (see orange box in Figure 2).

In the following section, the different kinetic models included in KiMoPack and the standard optimization routines for these models are described (see green box in Figure 2). Thereafter we describe how to create target models and present selected advanced models. Finally, we present a typical workflow including the optimization routine. Using the TA data set of Ru-dppz as an example, we show how the optimization settings are made and which options are available in addition to the standard optimization of the kinetic model, such as chirp or multiexperiment fit.

### Built-In Models and Global Lifetime Analysis

In KiMoPack, the time-resolved spectra (A_ab_) can be analyzed within the bi-linear approach, treating the profiles of the probe wavelength {*λ*_1_, λ_2_, *..., λ*_*n*_} and the times relative to the initial excitation (delay times) {*t*_1_, *t*_2_, ..., *t*_*m*_} independently.^22^ The time-resolved spectra are described by N independent spectral components with spectra  and their Φ parameter dependent, temporally developing concentration profiles  (see also the Supporting Information, Sections S1.1 and S1.2):
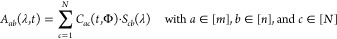
1The measured intensities (*A*_*a*b_) are described by the product of time-dependent concentration *C*_*ac*_(*t*,Φ) of each component and their corresponding (differential) spectral responses *S*_*cb*_(λ). The refined parameter together with the model function (rate equations) form the temporally developing concentration profiles (*C*_*ac*_) and describe the kinetic development of the system.

For this purpose, KiMoPack provides three internal kinetic models, namely exponential, consecutive, and full_consecutive (see dashed box in Figure 2), which allow a flexible adaption of the number of parameters (Φ) and the inclusion of background and non-decaying components (see documentation section *Fitting*(55)). In the first step of the optimization routine, time-dependent concentration profiles *C*_*ac*_ (*a* ∈ [*m*] and *c* ∈ [*N*]) are created depending on the kinetic model (see green box in Figure 2).

**exponential:** In this model all components *N* are taken to decay independent to each other in *parallel*. Hence, this model approximates the data by *N* independent exponential decays, namely *C*_*ac*_^′^ = exp(−*k*_c_ × *t*_a_). For each component, the exponential decay is convoluted with a symmetric Gaussian-shape response function (IRF), which reads as *C*_*a*c_ = *C*_*ac*_^′^ ⊗ IRF (see dashed green box in Figure 2).

**consecutive** and **full_consecutive**: These models assume that initially one excited state is populated, which decays unbranched and unidirectional (*A* → *B* → ··· → *N*). Thus, the decay of the initial component causes the population of a next component that turns to the following and so on. That is why this kinetic model is often referred as *sequential*. As a result, the concentration matrix is composed of both single exponential decays (describing the concentration profile of the initial state *A*) and weighted sum of exponential functions for the other *N* – 1 components that account for the population caused from the preceding component and reads as *C_ac_* =  (see definition of b_cd_ in the Supporting Information Section S1.2). The full_consecutive model is formed by this stepwise integrated differential equation. In the consecutive model the rate parameters are refined using the exponential model. In the final step, the consecutive differential equations are then formed with these optimized parameters and integrated for the extraction of the spectra. This approach is significantly faster and in many cases results in near equivalent solutions.

It should be emphasized that only the creation of *C*_*ac*_ depends on the particular kinetic model (kinetic parameters, such as rates, start-time, and resolution), while the subsequent steps of the optimization routine are identical for all models (built-in and user-defined target models). Once the concentration profiles (*C*_*ac*_) are created, the spectral components (*S*_*cb*_), i.e., decay associated spectra (DAS) for exponential models or species associated spectra (SAS) for all other models, are calculated unless they are externally provided. The calculated spectra *S*_*cb*_ are the solution of the linear matrix equation *C*_*ac*_ × *S*_*cb*_ = *A*_*ab*_, where *C*_*ac*_ × *S*_*cb*_ corresponds to the calculated fit data set (*AC_ab_*). In the last step, the measured and calculated data sets are compared (*AE_ab_* = *A_ab_* – *AC_ab_*) and the metric ∥*AE*_*ab*_∥^2^ is used as input in the optimization algorithm to determine if the termination conditions are reached. For each optimization step the parameters are updated and all previously described steps, calculation of *C*_*ab*_, *S*_*cb*_, *AE*_*ab*_, and the fit-error (∥*AE*_*ab*_∥^2^) are repeated. The optimization uses primarily the Nelder–Mead parameter optimization^20,56^ implemented in the minimize function of Scipy^57^ as a standard setting. Some advanced optimization routines that can overcome the trapping of the minimization in local minima (e.g., AmpGo^58^) are implemented and can be used in addition to the very important careful selection of starting parameter and flexible use of constraints to achieve a global minimum.

### Target and Advanced Kinetic Models

For more complex decay cascades, a target analysis strategy is required, where a specific model that combines, e.g., parallel and sequential reaction steps, is used to fit the data.^59−64^ As such kinetic schemes are commonly based on *a priori* knowledge of the system, this type of analysis is referred to as *target analysis*, where the target is to describe the real concentrations of the single components. In KiMoPack such target models are typically defined via kinetic rate equations (differential equations) or through other suitable functions (e.g., a measured instrument response function). For creating the time-dependent concentration profiles *C*_*ac*_ (*a* ∈ [*m*] and *c* ∈ [*N*]) for each component, which is the first step in the optimization routine (see global analysis section), a pulse profile and the differential equations are numerically sampled. Subsequently, the SAS (*S*_*cb*_), the fit data set (*AC*_*ab*_), and the difference between the measured and calculated data set *AE_ab_* = *A_ab_* – *AC*_*ab*_) are computed as described above (see section global analysis and green box in Figure 2).

KiMoPack was developed during continuous active use in several research groups and projects.^6,37,65−77^ This motivated the development of advanced modeling capabilities as are required by challenging samples and complex mechanisms, as well as the need to demonstrate the validity of the kinetic modeling approaches. Many analysis approaches simply use the implemented functionality in unexpected ways. Examples that might be of interest to the reader are, e.g., that single wavelength kinetics can be concatenated to a matrix or fitted separately or that multiple data sets (of the same or different techniques) can be fitted simultaneously using the same or a freely varying SAS. Other good analysis practices include testing of the (locked) initial guess parameter before refinement, the careful selection of the to be optimized parameters and limits, and the estimation of confidence intervals. In the following, we will present and discuss some scenarios that we have encountered in our analysis that go beyond the combination of consecutive and parallel processes.

#### Analysis of Nonlinear (Higher Order) Processes

When studying solid materials or molecules in sufficiently high concentration or at high excitation densities using time-resolved spectroscopy, nonlinear processes are often found. Typical examples for such processes are two photon or excited state absorption and annihilation/recombination processes. The observed rates or models do then depend on, e.g., the fluence of the excitation. In the provided examples the excited molecule fraction generated by sampling a pulse shape, which is given a fluence dependent amplitude. A nonlinear process would then be expressed in the usual way as, e.g.,  where [Y] represents the concentration of excited units and α is an exponent that represents the order of a process. This approach is particularly powerful if multiple data sets with different fluences/concentrations are analyzed simultaneously using the multi_project option in the global analysis.

#### Distributed-Rate Model Analysis

Distributed-rate models are an important approach for the description of natural systems that have a distribution of activation energies. Examples for systems like this are light induced polymer-folding or dynamics in polymer-based solar cells.^78,79^ The idea behind the modeling is that a distributed species is created and subdivided into smaller units. Then the dynamics of each unit is tracked separately in an independent matrix. In the returned concentration matrix these units are recombined into a single species with the same spectral feature. The system is then described (and optimized) with the parameters of the distribution. In the case of the example, the Gaussian distribution has a central rate constant and a width. We found that this approach often not only replaces several exponential decays (that would be required to explain the observed rates) but also represents a much more realistic description of the system.^78,79^

#### Spectroelectrochemistry

In spectroelectrochemistry, a fraction of the substances measured in the beampath are changed by a potential applied between two electrodes. Depending on the precise conditions, the absolute spectral changes that are induced can be very small, making the measurement challenging. In a recent experiment, we were unable to use a standard spectroelectrochemistry cell and instead focused a conventional lightsource onto a small round gold electrode and collected the reflected light using a fiber spectrometer.^13^ The electrode was part of a three electrode measurement system with which we performed repeated cyclovoltaic measurements. The measured current (compensated for a number of effects) was then read into KiMoPack as an indicator for the number of oxidized/reduced molecules and used as a model for the global analysis. Using the information obtained from the potentiostat allowed us to extract the spectrum of the transient species and perform further analysis based upon these spectra.

The example file, namely function_library.py, is provided in the Supporting Information and contains documented code for a simple sequential model, a model that includes a (laser) power-dependent dynamics and a distributed-rate model. Moreover, these models are included and documented in the advanced modeling workflow tool that can be found on the Github page^30^ of KiMoPack and as a locked release on zenodo.^31^

### Kinetic Modeling Workflow

In the following, a typical workflow for the global analysis of TA data with different kinetic models is described, using the TA data of Ru-dppz collected in DCM, ACN, and H_2_O as example. After preprocessing of the data we determine a first guess on the number and position of the main temporal components in the spectra. This visual inspection using the functions Plot_RAW() or Plot_Interactive() is often combined with a model-free matrix factorization technique, such as SVD, to explore the number of spectrally and temporally independent components.^22^ The number and magnitude of the singular values is an indication about the importance of the independent components in the data and often a cutoff value can be chosen. Other useful tools include 2D-correlation spectroscopy (2D-COS)^37^ that can use the preprocessed data as input.

**Figure 3:**
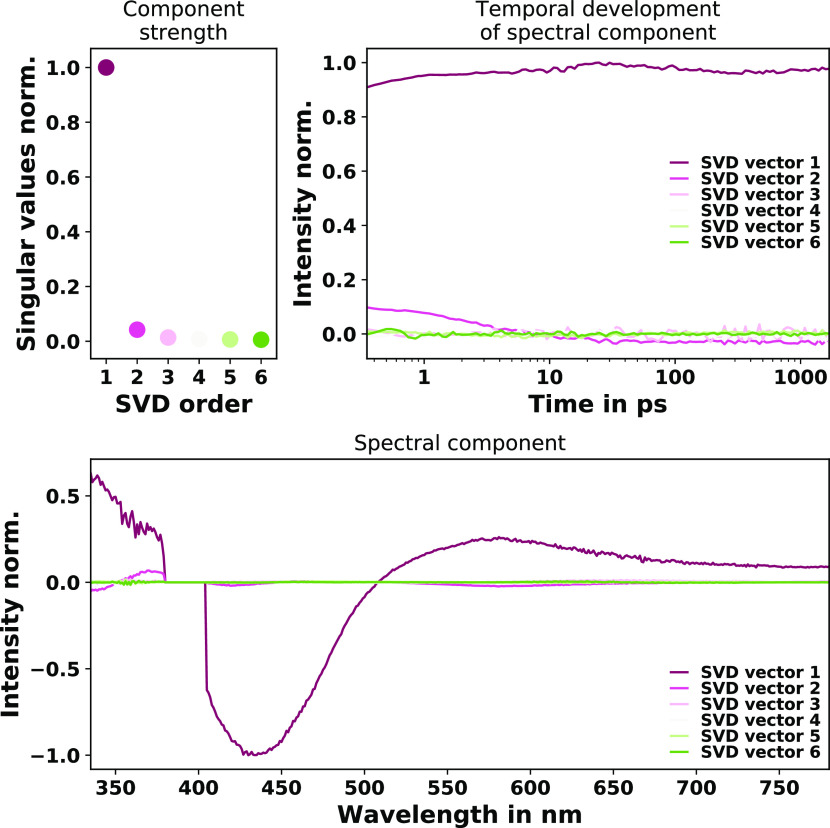
Singular value decomposition of the TA-matrix of Ru-dppz collected upon 400 nm excitation in acetonitrile. The plots show the singular values and respective spectral and temporal singular vectors for the first six singular components.

In the example of TA data of Ru-dppz collected in ACN at 400 nm excitation shown in Figure 3, the singular values and the shape of the singular vectors of the SVD indicate that the TA matrix most likely can be described by three main components, with one being dominant, and the characteristic times lie in the range between 1 and 2 ps and between 100 and 200 ps and contain one long-lived component (>2 ns). These initial insights are consistent with *a priori* knowledge about the photoinduced dynamics of Ru-dppz in ACN, indicating that the excited state dynamics can be described with three characteristic rate-constants.^46^ The next step is to assume three independent decay processes with the initial characteristic rates *k*_0_, *k*_1_, and *k*_2_. (note: the standard numbering starts at “0” in python, so *k*_0_ is the first decay component and the rates are linked to the characteristic times in the usual manner as *k* = 1/time). The fitting function (Fit_Global) uses the parameter control options of lmfit.^80^ For this purpose, each individual time constant is created as a parameter with starting value (e.g., ’k0′, value=1/2, vary=True) and passed into the corresponding lmfit parameter object (ta.par=lmfit.Parameters()). The optional flags vary=True/False or the setting of constraints using e.g., max=0.5 allows a flexible and controlled refinement, see the documentation section *Setting of Fit parameter* for more details.^55^

Commonly, the analysis starts by employing the exponential model to refine the set of initial parameters. The respective model definition and global analysis read as ta.mod=’exponential’ and ta.Fit_Global(). This usually serves as a useful starting point for subsequent modeling with more complex models, such as consecutive or target models.

**Figure 4:**
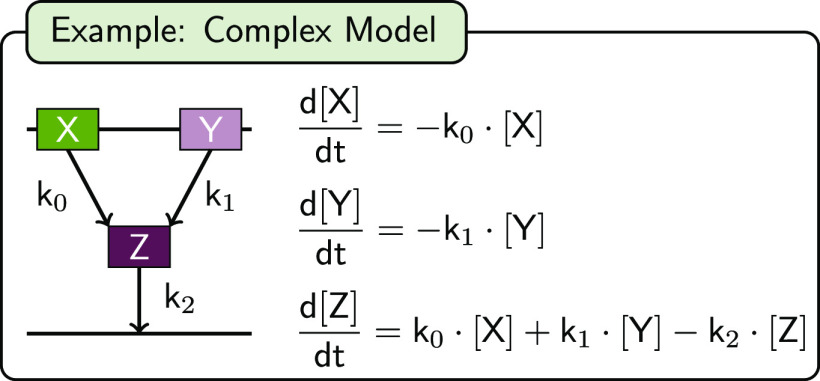
Schematic illustration of an user-defined (tutorial) model. In this model, the states X and Y are initially populated and the decay independently and irreversibly with the decay rate *k*_0_ and *k*_1_, respectively, finally populating state Z. This state irreversibly decays back to the ground state with the rate-constants *k*_2_. The corresponding rate equations for the concentrations of X, Y, and Z are displayed.

In the tutorial we assume as target model that two excited states (X and Y) are populated upon photoexcitation. In this model we sample a Gaussian peak function to generate X and Y and thus generate a broadened instrument response function. Those states independently and irreversibly decay with the rate constants *k*_0_ and *k*_1_, respectively, populating the excited state Z. Ultimately, Z decays back to the ground state with *k*_2_. The respective model sketch and rate equations for X, Y, and Z are summarized in Figure 4. The example code for the definition of the mentioned kinetic model and its application can be found in the tutorial notebook (cf. 02_KiMoPack_Fitting-2.ipynb). In all manual models the column labels of each species *N* can have arbitrary names (e.g., ’MLCT-hot’ or ’IL’), which are then used as plot labels.

The optimized parameter set can be evaluated by performing a confidence interval estimation using the switch confidence_level. This evaluation is different from the usual covariance based arguments in that it compares the continuously reoptimized model against the statistical fluctuation from the measured data.^20^ The confidence interval for each parameter is estimated by first calculating a statistically significant χ^2^ variation based on the scaled variance of the measured matrix, the number of parameters and the targeted confidence interval using a *F*-test (cf. Supporting Information, Section S1.3).^20^ Then each variable parameter is increased and decreased until the calculated χ^2^ reaches the so defined threshold, all other flexible parameters are simultaneously reoptimized for each step. The calculated confidence interval is thus the maximum variation on the multidimensional parameter surface and a narrow confidence interval is an indication of a model in which none of the other parameters cancompensate for this variation.

At any time, the preprocessing and model parameter can be changed, limited, or adapted to find a stable solution. We define and discuss criteria of a stable solution a little bit later in this paper. Tools that can help in this search are, e.g., the refinement of the arrival time correction (fit_chirp), or the use of advanced global optimizing algorithms. Currently, the adaptive memory programming for constrained global optimization^81^ (AMPGO, use_ampgo) is employed in KiMoPack to automatically explore the data for different local minima.

## Collecting and Plotting Routine

Data can be (interactively) plotted and exported (in form of images or tabular data) at any time during the analysis. The usual examples are prior or after preprocessing or optimization of the fit parameters. As each analysis problem might require a different view on the data, we use a function to generate a number of plots automatically but provide options to select and shape a favorite representation. Typical examples are illustrated in the orange box in Figure 2.

### Plotting of Raw- and Preprocessed Data

Generally, three different types of plots are used to visualize the data: (i) 2D-contour plots, (ii) kinetic traces, and (iii) transient spectra. In the 2D-map, the value of the measurement (e.g., differential absorption or fluorescence intensity) is depicted as a function of the time delay (abscissa) and the probe wavelengths (ordinate). The kinetic traces and transient spectra are horizontal or vertical slices of this data. The function Plot_Interactive allows the interactive visualization of a transient spectrum and its kinetics by cursor placement on the 2D-map. The Plot_RAW-function uses specified wavelength and times to generate an overview of the interesting information and generate publication ready plots. A wide range of arguments in the TA object can alter the appearance of the plots and the shape of the data. The example and template *Jupyter* notebooks include a selection of the commonly used options that, e.g., suppress the pump scattered light or the artifacts that stem from cross-phase modulation around time-zero (*t* = 0). Commonly, these parameters are adjusted at the beginning of an analysis project for a specific purpose, refined later if needed and duplicating for similar analysis projects. For further information, see the examples and the documentation section *Shaping of data*.^54^

### Plotting of Fit Results

**Figure 5:**
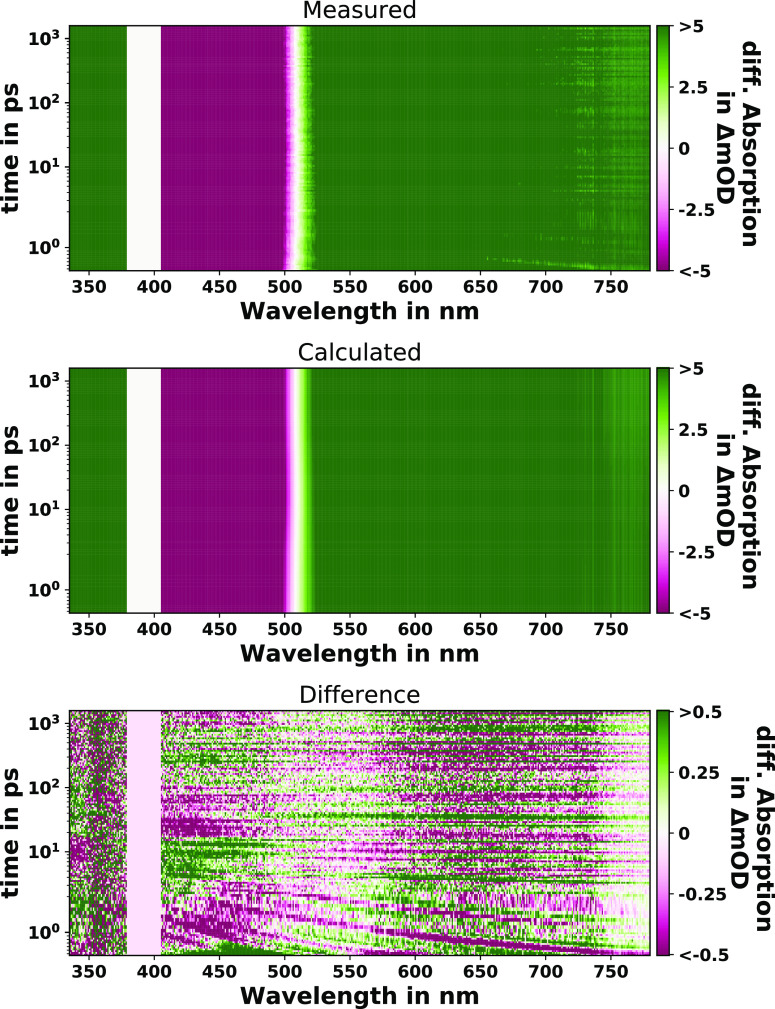
Contour plots of the preprocessed (measured: *A*_*ab*_) and modeled (calculated: *AC*_*ab*_) fs-TA data of Ru-dppz in acetonitrile (ACN) in the time-delay range of 350 fs to 1800 ps and probe wavelength range of 330 to 780 nm upon photoexcitation at 400 nm. The bottom plot shows the 10-fold amplified difference of the measured and calculated data (*AE*_*ab*_) enabling an inspection of the overall fit quality.

The quality of the fit can be evaluated based on the fit parameters returned by the Fit_Global function as well as the plot(s) that are created by the Plot_fit_output or Plot_Interactive function. Plot_fit_output generates six plots that contain the most often used indicators. (i) 2D-contour plots of the measured, modeled and difference matrix (see Figure 5), (ii) the integrated kinetic traces, (iii) the selected kinetic traces, (iv) the selected spectra, and (v) the transient spectra (DAS/SAS) as well as (vi) the temporal profiles of each component. A step-by-step guide is shown in the tutorial notebook and further information in documentation section *Plotting functions*.^82^

### Evaluation of the Quality of a Model Representation (Fit Quality)

We will try to formulate in this section which parameter we employ to evaluate the quality of a model representation. The reader will note that the reduced χ^2^ is consciously not included. It includes the number of parameters and is as such mainly useful in comparing descriptions, but challenged through the continuous optimization of the spectra (and the large number of parameters in the spectra). Internally the similar metric ∥*A_ab_* – *AC*_*ab*_∥^2^ is used to evaluate the optimization progression. Instead, several indicators should be combined to define a sufficiently good description of a data set and we recommend the following criteria:

(1) Closeness of Fit: The coefficient of determination, also called the *R*^2^ parameter, is close to 1.0. The *R*^2^ compares the residual between model and fit to the variance of the data. If a residual matrix (see bottom panel in Figure 5) only shows values close to zero in all regions, without any visible structure, this is a good description and will lead to an *R*^2^ value close to 1.0. In this example periodic structure or specific spectral regions that are not well described (also note the different color scale to estimate the magnitude). In the optimization process, all plots from Plot_fit_output should be used to estimate this criteria in addition to the *R*^2^ value.
(2) Precision: A narrow(er) confidence interval often indicates a good/better model.
(3) DAS/SAS: None of the DAS/SAS are mirror images of each other (indicating linear compensating), and specific features are verifiable with other spectroscopic techniques (e.g., spectrophotometric, acid/base titration, or spectroelectrochemistry).
(4) Sensitivity: The fit-parameters are insensitive to small changes in the preprocessing parameters.
(5) Stability: The initial guesses of the starting parameters do not strongly influence the fitted parameters.
(6) Global Minimum: Other minima using the same model and a feasible parameter space represent the data worse (under consideration of the error margins)
(7) Defensible Model: The proposed model must be physically correct and consistent with other techniques and chemical principles. To verify or constrain a model is good scientific praxis.
(8) Simpler Models: In general, all models that fulfill the criteria 1–7 should be discussed, and external arguments should be used to disregard other feasible descriptions. Finding/defining external constraints often helps to minimize the number.

### Data Export and Project Saving

**Figure 6:**
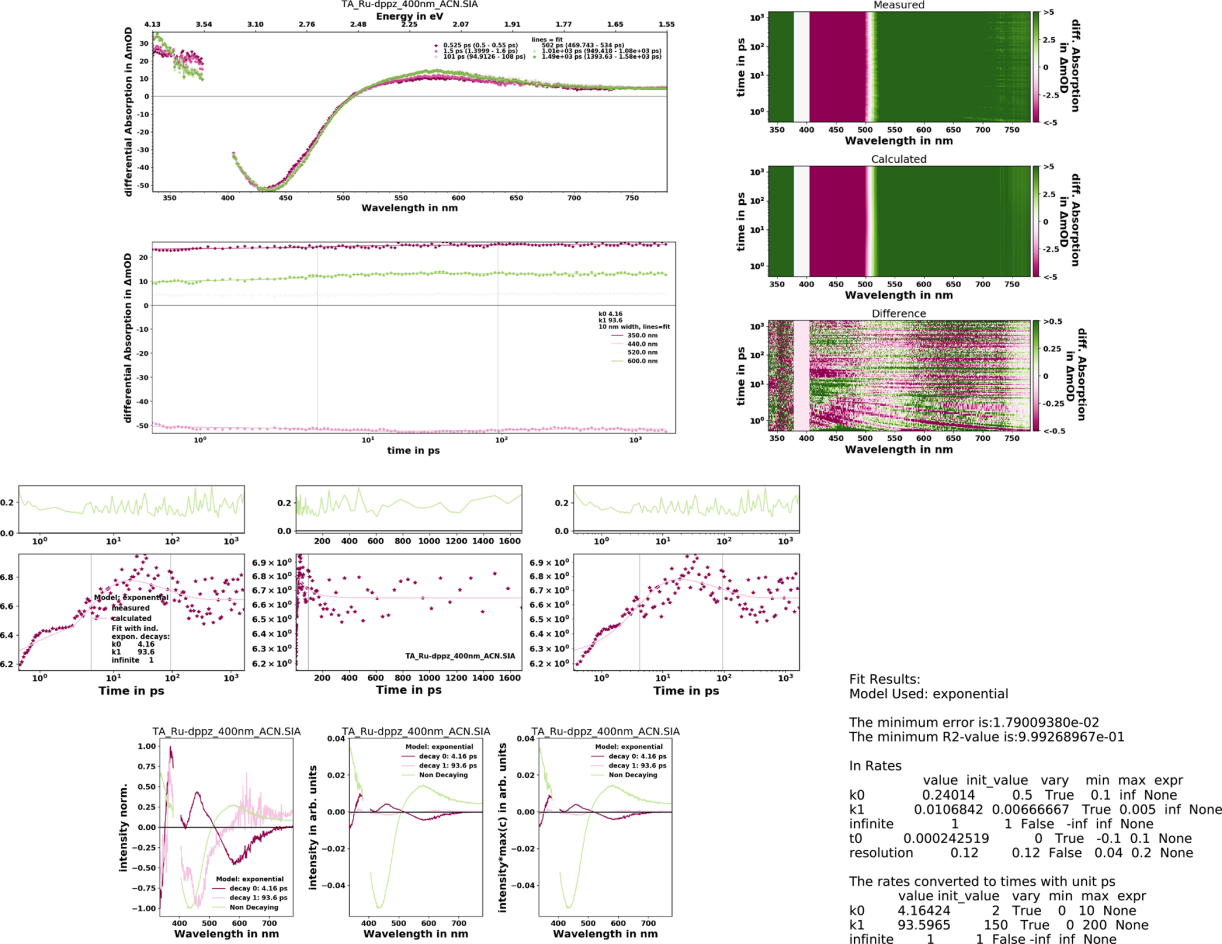
KiMoPack-generated summary of the preprocessed TA data and the fit results of Ru-dppz in acetonitrile (ACN) upon 400 nm excitation.

The export of preprocessed data (Save_Data) and fit results in the form of images, tabular files and as report summary are important for extending the functionality to other tools. All graphs can be saved as single files or via the function Save_Plots all at once. Particularly useful for creating fast reports is the function Save_Powerpoint that creates one summary file as *.pptx, *.pdf, *.svg or two *.png files, containing all relevant graphs and fit results. (Figure 6). The whole analysis project including all relevant data and fit results can be saved and recalled as *hdf5* file. These summary files are particularly useful for the comparison of several data sets, e.g., those collected in different solvents, at different excitation wavelengths, and various quencher concentrations or the comparison of different analysis strategies. A detailed description can be found in the documentation sections *Plotting functions*(82) and *Data Export and Project Saving*(83)

### Comparison of Data Sets and Fit Results

To compare data sets and fit results is an important point of most analysis. It does contribute to understand the influence of different conditions onto the processes. During the comparison of data often the need for flexible normalization of different data sets is challenging. Three functions were designed to simplify the comparison of data. Transient spectra at defined delay times are shown with Compare_at_time that also allows the inclusion of external spectra. Kinetic traces at selected wavelengths are displayed with Compare_at_wave and the modeled spectra (DAS or SAS) are shown with Compare_DAC. The first two functions include a flexible normalization procedure that integrates the intensity in a defined spectral–temporal window and divides all spectra by this value. This permits, for example, normalization to the intensity of the ground state bleach signals to compare measurements with, i.e., different sample concentrations, solvents, or excitation intensities or the normalization to an excited state absorption to extract the relative efficiency of photo product formation. Spectra (DAS, SAS, or TA spectra) can be compared to external data, such as the absorption spectra of electrochemically modified species, photoproducts or simulated spectra, which has been proven to be tremendously helpful in evaluating complex data.^5,84,85^ The *Jupyter* notebook 03_KiMoPack_CompareFit.ipynb demonstrates the comparison procedure using the TA data of Ru-dppz collected in DCM, ACN, and H_2_O.

### Interpretation of Fit Results

Unfortunately there is no universal procedure/recipe for the interpretation of fit results. The particular challenges often lay in the assignment of photochemical and photophysical processes occurring with a characteristic rate-constant *k*_*c*_. The combination of lifetimes together with the process oriented DAS and the species selective SAS can however often be used to indicated a particular process, particularly if they can be compared to, e.g., spectro-electrochemical results or if the interpretation is supported by multiple techniques.

**Figure 7:**
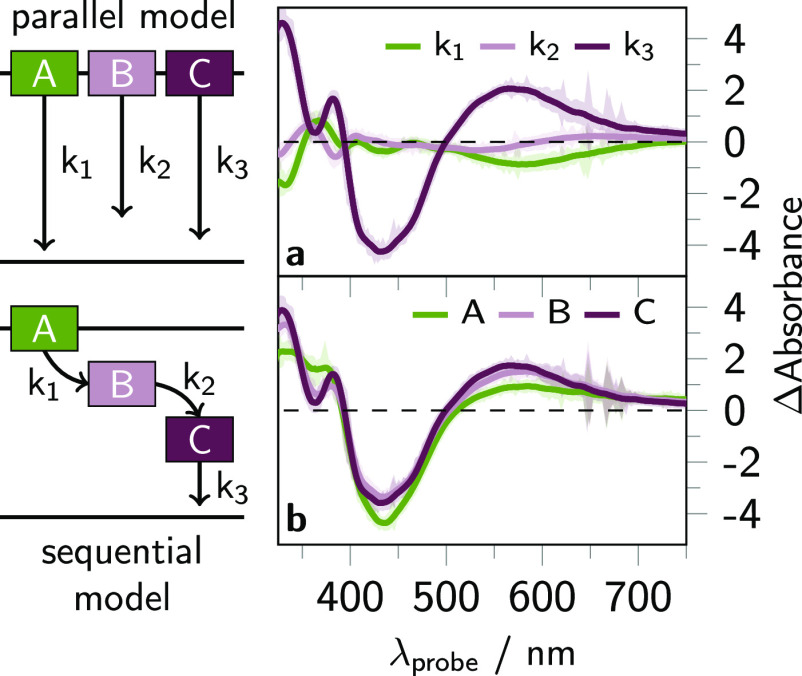
Schematic representation of a parallel and sequential model involving three different species, namely A (^3^MLCT_prox_), B (^3^MLCT_dist_), and C (^3^MLCT_phen_). Decay associated spectra (DAS, a) and species associated spectra (SAS, b) obtained from global analysis of the transient absorption (TA) data of Ru-dppz collected upon 400 nm excitation in acetonitrile (ACN) within the exponential (giving DAS) and consecutive model (giving SAS).

We found that new user of transient absorption find the difference between DAS and SAS particularly challenging to understand and would like to use the analysis of one process in the model complex Ru-dppz to highlight the difference. A detailed description of the excited state processes of the model complex Ru-dppz can be found in the literature (see also Figure 1).^38−40,42−46^ We use exemplary the interligand hopping process to highlight the difference and rely on that certain feature of the SAS have been identified as characteristic ligand-to-metal charge-transfer or ππ*-absorption bands in prior work. In this hopping process the electron density is shifted from the tbbpy ligand to the dppz ligand sphere,^46^ and this shift is manifested in the DAS and SAS (see Figure 7). Considering the DAS, the interligand hopping process is reflected in the DAS associated with *k*_1_ as follows: The excited state absorption (ESA) centered around 380 nm, which is assigned to states with excess electron density on the tbbpy ligands, decreases (positive signal region), while the ESA for phz-centered states at 340 and 590 nm increases (negative signal region). Similarly, this interligand hopping process is manifested in the SAS when the spectra associated with the species A and B are compared: The latter spectrum (A) stems from a phenazine-centered MLCT state (^3^MLCT_phz_) showing prominent ESA maxima at 340 and 590 nm, which can be assigned, e.g., via comparison with UV–vis spectroelectrochemical data.^86^ The A-associated spectrum exhibits the characteristics typically observed for Ru(II) polypyridine-type complexes, namely comparably strong ground state bleach (GSB) between 400 and 500 nm accompanied by a strong and broad as well as a unstructured and flat ESA band between 340 and 400 nm and above 500 nm, respectively. Consequently, the first species can be assigned to a state with excess electron density in the proximal ligand sphere (^3^MLCT_prox_: ^3^MLCT_tbbpy_, ^3^MLCT_phen_).

## Conclusion

The deeper analysis of spectrally- and time-resolved data usually required commercial software or extended programming knowledge, as many open-source software just provide fitting routines for simple kinetic schemes. We developed a flexible python-based toolbox, named KiMoPack, which allows global and target modeling of transient data with implemented and user-defined models without the prerequisite of extensive simulation/programming knowledge. Through this introduction, the tutorials and workflow sheets the intuitive functions of KiMoPack were demonstrated at the example of transient absorption data of Ru-dppz recorded in different solvents. Generally, the tool can be used for the analysis of any transient/time-resolved spectra, i.e., optical or X-ray emission or Raman spectroscopy data sets, and can show through its open-source and modular nature the potential for further extensions by the community. Recently, KiMoPack was used for the modeling transient absorption, X-ray, time-resolved emission, spectroelectrochemistry, and photocatalysis data.

In summary, the work demonstrates the use of KiMoPack to analyze complex spectroscopic data, and we believe that this tool offers ways to simplify and combine the standard analysis routine with the improved objectivity of performing all preprocessing, fitting, plotting, reporting, and more analysis steps in a single powerful analysis tool.

## Supporting Information Available

Detailed explanation of the model construction, error analysis, data export, and a summary of the tutorials (PDF)
File containing *Jupyter* notebook demonstrating the general workflow of KiMoPack to analyze TA data of Ru-dppz collected in DCM, ACN and H_2_O employing two *built-in* kinetic models (parallel and sequential model), *Jupyter* notebook showing how to define and implement a user-defined kinetic model in the global fit using Ru-dppz in acetonitrile as an example, *Jupyter* notebook using the example of Ru-dppz to show how TA data recorded in different solvents, namely DCM, ACN, and H_2_O, can be (visually) compared to each other and external spectra, i.e., steady-state and spectroelectrochemical absorption data, *Jupyter* notebook showing how individual TA scans of Ru-dppz (in ACN, DCM, or H_2_O) can be selected and averaged using an interactive plot, a python module file that contains the documented definitions of three function for the generation of a consecutative model, a non linear power dependent model and a model with distributed rate, folder containing the raw data used for the tutorials, where the data are subdivided into a specific folder per tutorial, and a folder containing the images rendered in the tutorial notebooks (ZIP)
