# Supplementary material for: KiMoPack: A python Package for Kinetic Modeling of the Chemical Mechanism
Source: J Phys Chem A. 2022 Jun 14;126(25):4087–99. doi: 10.1021/acs.jpca.2c00907 (PMC9251768; doi:10.1021/acs.jpca.2c00907)
Supplement: Supplementary file 1 — jp2c00907_si_001.pdf [file jp2c00907_si_001.pdf]

Supporting Information for Publication

KiMoPack – A python Package for Kinetic Modelling of Chemical Mechanism

Carolin Müller[1,2], Torbjörn Pascher[3], Axl Eriksson[3], Pavel Chabera[3], and Jens Uhlig[3,*]

[1]Friedrich Schiller University, Institute for Physical Chemistry, Helmholtzweg 4, 07743 Jena, Germany
[2]Leibniz Institute of Photonic Technology (IPHT) Jena, Albert-Einstein-Str. 9, 07745 Jena, Germany
[3]Lund University, Department of Chemical Physics, SE-22100 Lund, Sweden
[*]Corresponding author: jens.uhlig@chemphys.lu.se

Contents

1 **Explanation of the Build-in Models for Global Lifetime Analysis** **S 3**
1.1 Parallel Model (`exponential`) . . . . . S 3
1.2 Sequential Model (`full_consecutive`) . . . . . S 4

2 **Error Analysis** **S 7**

3 **Data Export** **S 8**

4 **Tutorials** **S 9**
4.0.1 The Model Substance - `Ru-dppz` . . . . . S 9
4.0.2 Working with *Jupyter* Notebooks . . . . . S 9
4.1 Tutorial `01_KiMoPack_Fitting-1.ipynb` . . . . . S 10
4.1.1 Load Data . . . . . S 10
4.1.2 Background correction and filtering . . . . . S 11
4.1.3 Arrival time correction . . . . . S 11
4.1.4 Plot the standard corrected data . . . . . S 11
4.1.5 Plot Singular Value Decomposition (SVD) Results . . . . . S 13
4.1.6 Fitting of the Data . . . . . S 13
4.1.7 Plot Fit Results . . . . . S 16
4.1.8 Save Results . . . . . S 18
4.2 Tutorial `02_KiMoPack_Fitting-2.ipynb` . . . . . S 22
4.2.1 Import data . . . . . S 22
4.2.2 Standard corrections . . . . . S 22
4.2.3 Plot pre-processed data . . . . . S 22
4.2.4 Fitting of the data . . . . . S 25
4.2.5 Repeat the global analysis to estimate the errors . . . . . S 26
4.2.6 Plotting of error Analysis (advanced handling) . . . . . S 27
4.2.7 Plotting of error Analysis (advanced handling) . . . . . S 28
4.2.8 Shaping of the plot . . . . . S 30
4.2.9 Target analysis – propose a model . . . . . S 30
4.2.10 Define own model function . . . . . S 31
4.2.11 Define fitting parameters . . . . . S 32
4.2.12 Fitting of the Data - Kinetic Modeling (Ru-dppz Model) . . . . . S 33
4.3 Tutorial `03_KiMoPack_CompareFit.ipynb` . . . . . S 35
4.3.1 Saving and Loading of Projects . . . . . S 35
4.4 Tutorial `04_KiMoPack_ScanHandling.ipynb` . . . . . S 41

1 Explanation of the Build-in Models for Global Lifetime Analysis

In **KiMoPack** the following three models are in-built:

`exponential` | In this model all components (N) are taken to decay independent to each other in *parallel*. Hence, this model approximates the data by N independent exponential decays, namely $C_{ac} = \exp(-k_c \times \mathrm{t}_a)$. This exponential decay is convoluted for each component with a symmetric Gaussian-shape response function. The extracted spectra are the pre-factors of the single exponential decays ($S_{cb} = a_{cb}$) and are commonly referred as decay associated spectra (DAS). For more details see section Parallel Model (`exponential`).

`consecutive`, `full_consecutive` | Those models assume that initially one excited state is populated, which decays unbranched and unidirectional ($\mathrm{A} \rightarrow \mathrm{B} \rightarrow \cdots \rightarrow \mathrm{N}$). Thus, the decay of the initial component causes the population of a next component that turns to the following and so on. That is why this kinetic model is often referred as *sequential*. As a result, the concentration matrix is composed of both single exponential decays (describing the concentration profile of the initial state X) and weighted sum of exponential functions for the other $\mathrm{N}-1$ components that account for the population caused from the preceding component. The `full_consecutive` model is formed by this step-wise integrated differential equation. The `consecutive` model uses the `exponential` model to determine the lifetimes and the `full_consecutive` model to calculate the species associated spectra (SAS). For more details see section Sequential Model (`full_consecutive`).

Details about the estimation of the errors of the fit parameters can be found in section Error Analysis
.

1.1 Parallel Model (`exponential`)

A typical time-resolved dataset is composed of intensities/signals collected at a set of n probe wavelengths $\{\lambda_1, \lambda_2, ..., \lambda_n\}$ and at m times relative to the instant of excitation $\{t_1, t_2, ..., t_m\}$.

Example: Parallel Model

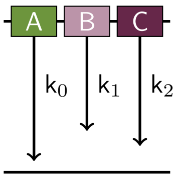

$$\frac{\mathrm{d[A]}}{\mathrm{dt}} = -\mathrm{k}_0 \cdot [\mathrm{A}]$$

$$\frac{\mathrm{d[B]}}{\mathrm{dt}} = -\mathrm{k}_1 \cdot [\mathrm{B}]$$

$$\frac{\mathrm{d[C]}}{\mathrm{dt}} = -\mathrm{k}_2 \cdot [\mathrm{C}]$$

```
ta.mod='exponential'
par=lmfit.Parameters()
par.add('k0',value=1/2.5,vary=True)
par.add('k1',value=1/150,vary=True)
par.add('k2',value=1/250000,vary=True)
```

Figure S1: *Left:* Schematic sketch of the parallel model describing the independent decay of the three initially excited states A, B and C with k_0, k_1 and k_2, respectively, populating the final states (A', B' and C') and the corresponding rate equations of the parallel occurring elementary reactions. *Right:* Minimal python code example for using such a parallel model from the internal kinetic models.

The parallel model description is based on a number of initially photoexcited states N, that are taken to decay independently from each other following first order kinetics. Hence, the concentration matrix (C) for each single compartment $\{p_1, p_2, \ldots, p_N\}$ reads

$$\frac{\mathrm{d}p_c}{\mathrm{d}t} = -k_c \cdot p_c \qquad \text{with } c \in [N]\,. \tag{1}$$

Initially ($t = 0$), all compartments start decaying and thus it is required, that the starting concentrations of all compartments equal 1 ($p_1^0 = p_2^0 = \cdots = p_N^0 = 1$). Hence, the solution of the first order rate equations (1) yield an exponential decay with a characteristic rate-constant (k_c) for each compartment. Those exponential functions represent the elements of the concentration matrix within the parallel model, which reads as

$$C_{ac}^{\parallel} = \exp\left(-k_c \cdot t_a\right) \qquad \text{with } c \in [N],\ a \in [m] \tag{2}$$

If the width of the instrument-response function (IRF) is negligible, the parallel model for the ΔAbs transient absorption signals reads

$$\Delta A_{ab} = \sum_{c=1}^{N} C_{ac}^{\parallel} \cdot S_{cb}^{\parallel} \qquad \text{with } a \in [m], b \in [n] \,. \tag{3}$$

If the IRF is not negligible the exponential decay is convoluted with a Gaussian function (`gauss`), which reads as

$$\text{IRF} = \frac{1}{\sigma \cdot \sqrt{2 \cdot \pi}} \cdot \exp\left(-\frac{0.5 \cdot (t-\mu)^2}{\sigma^2}\right) \qquad \text{with } \mu \in [m] \tag{4}$$

and describes the IRF *via* a parameter for the location (time-zero, μ) and the width (σ). When employing the parallel fitting model (`exponential`), such a symmetric response function is formed for each compartment (N). The $S_{cb}^{\parallel}$ in equation (3) represent the estimated amplitudes $a_{cb}^{\parallel}$ of the exponential decays defined in equation (2) for each probe wavelength $\{\lambda_1, \lambda_2, \ldots, \lambda_n\}$, and thus, the so-called decay associated spectra (DAS) and read as

$$S_{cb}^{\parallel} = a_{cb}^{\parallel} \qquad \text{with } c \in [N], b \in [n] \,. \tag{5}$$

In connection with equation (2), negative contributions ($a_{cb}^{\parallel} < 0$) describe the rise and positive contributions ($a_{cb}^{\parallel} > 0$) the decay of ΔAbs signals.

1.2 Sequential Model (`full_consecutive`)

The sequential model assumes that initially a single excited state is populated, which in turn decays unbranched and unidirectional. In general, the rate equations for that model can be solved by

$$C_{ac}^{\uparrow} = \sum_{c=1}^{d} b_{cd} \cdot \exp\left(-k_c \cdot t_a\right) \qquad \text{with } d \in [N],\ a \in [m] \,, \tag{6}$$

where k_c is the decay rate of compartment c and b_{cd} are the amplitudes of the exponential decay, which are defined by Eq. (7). Thus, each compartment concentration is a linear combination of the exponential decays and the amplitudes b_{cd}.

$$b_{cd} = \prod_{e=1}^{d-1} k_e \cdot \left(\prod_{f=1, f\neq c}^{d} \left(k_f - k_c\right)\right)^{-1} \qquad \text{with } c, d \in [N]\,, b_{11} = 1, c \leq d. \tag{7}$$

With that, the sequential model for the time-resolved spectra (*e.g.*, transient absorption data) reads as

$$\Delta A_{ad} = \sum_{c=1}^{N} C_{ad}^{\uparrow} \cdot S_{db}^{\uparrow} \qquad \text{with } a \in [m], d \in [n] \,. \tag{8}$$

The species associated spectra (SAS) within the model $S^{\uparrow}$ are defined as

$$S^{\uparrow}_{db} = a^{\uparrow}_{db} \qquad \text{with } d \in [N] \,.b \in [n] \tag{9}$$

In a three component example, the first compartment (component A) is initially excited and irreversibly decays with the decay-rate k_0 to form component B, which is represented by the second compartment. This component B also decays irreversibly but with rate-constant k_1 thereby forming C, which decays with k_3 back to the ground state (A → B → C → GS). This kinetic scheme is depicted in Figure S2. The corresponding rate equation for the first compartment (initially excited state A) reads as

$$\frac{\mathrm{d}[\mathrm{A}]}{\mathrm{d}t} = -k_0 \cdot [\mathrm{A}] \,. \tag{10}$$

Example: Sequential Model

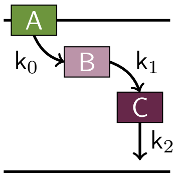

$$\frac{\mathrm{d[A]}}{\mathrm{dt}} = -\mathrm{k}_0 \cdot [\mathrm{A}]$$

$$\frac{\mathrm{d[B]}}{\mathrm{dt}} = \mathrm{k}_0 \cdot [\mathrm{A}] - \mathrm{k}_1 \cdot [\mathrm{B}]$$

$$\frac{\mathrm{d[C]}}{\mathrm{dt}} = \mathrm{k}_1 \cdot [\mathrm{B}] - \mathrm{k}_2 \cdot [\mathrm{C}]$$

```
ta.mod='consecutive'
par=lmfit.Parameters()
par.add('k0',value=1/2.5,vary=True)
par.add('k1',value=1/150,vary=True)
par.add('k2',value=1/250000,vary=True)
```

Figure S2: *Left:* Schematic sketch of a sequential model, in which the first compartment (A) is initially excited and irreversibly decays with the decay rate k_0 to form component B (second compartment). This component B also decays irreversibly but with the rate-constant k_1, thereby forming C, which decays with k_2, and the corresponding rate equations for the concentrations of A, B and C. *Right:* Minimal python code example for using such a parallel model from the internal kinetic models.

Since only the first compartment is initially excited, the initial conditions (t_0) for this case are $[\mathrm{A}]_0 = 1$ and $[\mathrm{B}]_0 = [\mathrm{C}]_0 = 0$. Hence, the decrease of [A] yields a first order rate equation (see Eq. 2). The concentrations of B and C are decaying as described by the following rate equations.

$$\frac{\mathrm{d}[\mathrm{B}]}{\mathrm{d}t} = k_0 \cdot [\mathrm{A}] - k_1 \cdot [\mathrm{B}] = k_0 \cdot \exp\left(-k_0 \cdot t\right) - k_1 \cdot [\mathrm{B}] \tag{11}$$

$$\frac{\mathrm{d}[\mathrm{C}]}{\mathrm{d}t} = k_1 \cdot [\mathrm{B}] - k_2 \cdot [\mathrm{C}] \tag{12}$$

Integration of 11 gives

$$[\mathrm{B}] = \frac{\mathrm{k}_0}{(\mathrm{k}_1 - \mathrm{k}_0)} \cdot \left[\exp\left(-\mathrm{k}_0 \cdot \mathrm{t}\right) - \exp\left(-\mathrm{k}_1 \cdot \mathrm{t}\right)\right] \,. \tag{13}$$

Substituting [B] in formula 12 with the expression 13 allows to solve the differential equation for [C] yielding

$$[\mathrm{C}] = \frac{k_0 \cdot k_1}{(k_1 - k_0)(k_2 - k_0)(k_2 - k_1)} \cdot \left[-(k_2 - k_1)\exp(-k_0 \cdot t) + (k_2 - k_1)(k_2 - k_0)\exp(-k_1 \cdot t) + (k_1 - k_0)\exp(-k_2 \cdot t)\right] \,. \tag{14}$$

Therefore, the concentration matrix of the sequential model ($\mathsf{C}^{\uparrow}_{\mathsf{ad}}$) reads as

$$\begin{pmatrix} e^{-k_1\cdot t_1} & b_{12}\cdot e^{-k_1\cdot t_1}+b_{22}\cdot e^{-k_2\cdot t_1} & b_{13}\cdot e^{-k_1\cdot t_1}+b_{23}\cdot e^{-k_2\cdot t_1}+b_{33}\cdot e^{-k_3\cdot t_1} \\ \vdots & \vdots & \vdots \\ e^{-k_1\cdot t_m} & b_{12}\cdot e^{-k_1\cdot t_m}+b_{22}\cdot e^{-k_2\cdot t_m} & b_{13}\cdot e^{-k_1\cdot t_m}+b_{23}\cdot e^{-k_2\cdot t_m}+b_{33}\cdot e^{-k_3\cdot t_m} \end{pmatrix} . \tag{15}$$

The respective concentrations in the sequential model (see Eq. 15) are a linear combination of mono-exponential, first-order decays. As a consequence, the $\mathsf{C}^{\uparrow}$ (*cf.* Eq. 15) can be expressed in terms of $\mathsf{C}^{\|}$ (*cf.* Eq. 2) *via*

$$C^{\uparrow} = C^{\|}\cdot\Phi \quad . \tag{16}$$

Within the three-compartment example, Φ reads as

$$\begin{pmatrix} 1 & b_{12} & b_{13} \\ 0 & b_{22} & b_{23} \\ 0 & 0 & b_{33} \end{pmatrix} \tag{17}$$

where the upper triangular matrix of A contains the elements b_{cd} as defined in equation (7). Combining equations (3), (8), (16) gives the relation between the DAS and SAS matrices:

$$C^{\|}\cdot S^{\|} = C^{\|}\cdot\Phi\cdot S^{\uparrow}$$

$$\leftrightarrow S^{\|} = S^{\uparrow}\cdot A^{T} \tag{18a}$$

$$\leftrightarrow S^{\uparrow} = S^{\|}\cdot\left(A^{-1}\right)^{T} \tag{18b}$$

Thus, the DAS are a linear combination of the SAS and *vice versa*: The j-th SAS is a linear combination of the j-th and the following DAS. For instance, the first SAS (at $t = 0$) is the sum of all DAS, and the final SAS is proportional (factor b_{33}^{-1}) to the final DAS.

2 Error Analysis

Estimating errors correctly is based on estimating the validity of the full set of optimized parameter. For this we use the *F*-statistics of a single or multiple combined datasets to define a cutoff value of the χ^2 at which we have to assume that the 0-Hypothesis (the two models with the parameters are the same) has to be rejected in favor of the alternative hypothesis (the models with the parameters are different). Into the *F*-statistics go all parameter that influence the quality of the modelling. These are all free parameters of the model (*e.g.*, the kinetic parameter) plus for each spectral component that is extracted one parameter for each wavelength that is fitted. So the number of parameters is most strongly influenced by the number of spectral species that are to be extracted.

number of fitted parameter=
number of species · number of spectral points + number of (kinetic) parameters

The *F*-statistics defines a value that is called *free points* that is calculated as

number of *free points* =
total measured points – fitted parameters

The *total spectral points* are all the points that we after pre-treatment of the data still have in the dataset:

total measured points =
number of spectral points · number of time points (for each dataset included)

At the cutoff value the χ^2 of the parameter set is then so much larger than the minimum χ^2 that this cannot be explained statistically anymore. We can now define a target quality, meaning, what fraction of the data lays inside and outside of the confidence environment in the definition 100% · `target_quality`. For each (varied) kinetic parameter we now perform a separate optimization, that attempts to find the upper and lower bound at which the total error of the re-optimized globally fitted results reaches this cutoff value. All other (varied) parameters, including the spectral intensities are re-optimized for each step of this test. This means that the error calculation calls the global optimization routine (with one parameter less) many times and might run for long time, typically 50–100 times longer than a single optimization. The width of the extracted confidence interval is however a very good estimation for the quality of the model vs. the measured data quality. Practically the error estimation is triggered by adding the `confidence_interval` option in the `Fit_Global` function. The fit results will then contain the confidence intervals.

Plotting the confidence intervals into the kinetics is challenging, as a large number of combinations might need to be plotted. The user might however choose the limits from the parameter that are representative, select the `vary=False` option for all parameter and create such a plot using the `Compare_at_wave` or `Compare_at_time` functions (see section Tutorials).

3 Data Export

When the data is exported using the `Save_data` function with the settings `save_RAW=True` and `save_Fit=True` the following twelve files are written to the specified folder.

file-endings and description of saved files containing pre-processed/raw data:

***_chirp_corrected_raw_matrix.dat** chirp corrected 2D-matrix at all studied delay times and probe wavelengths

***_matrix used as fit input.dat** 2D-matrix at probe wavelengths and delay times as set by `bordercut` and `timelimits`

***_chirp_corrected_RAW_Spectra.dat** transient spectra at selected delay times (`rel_time`)

***_measured_spectra.dat** transient spectra at all studied delay times

***_chirp_corrected_RAW_kinetics.dat** kinetic traces at selected probe wavelengths (`rel_wave`)

***_measured_kinetics.dat** kinetic traces at all studied probe wavelengths

File-endings and description of saved files containing fitted data:

***_matrix calculated during fit.dat** fitted 2D-matrix at probe avelengths and delay times as set by `bordercut` and `timelimits`

***_error_matrix calculated during fit.dat** difference of fitted and pre-processed 2D-matrix

***_fitted_spectra.dat** fitted transient spectra at selected delay times (`rel_time`)

***_fitted_kinetics.dat** fitted kinetic traces at selected probe wavelengths (`rel_wave`)

***_fit_results_parameter.par** summary of starting and optimized fit paramaters

***_DAS-SAS.dat** decay-(`mod='exponential'`) or species associated spectra (`mod='consecutive'`)

4 Tutorials

4.0.1 The Model Substance - Ru-dppz

Several spectroscopic studies revealed, that this reminiscent property originates from the two acceptor orbitals of the dppz ligand, namely the phenanthroline (phen) and phenazine (phz) moiety.[1, 2] This yields the existence of two low-lying MLCT states in the excited state manifold, *i.e.*, a bright (emissive) $^3MLCT_{phen}$ and a dark (non-emissive) $^3MLCT_{phz}$ state.[3, 4, 5, 6, 7] The population ratio between those two states can be altered by changing the solvent. The population of the dark state is reduced, *e.g.* in organic solvents, but increased in aqueous solution as the energy of the phz-centered state is sufficiently lowered by the interaction with surrounding water molecules.[3, 4, 6, 8, 9, 10]

For example, in ACN solution it is known that the light-induced dynamics occurs exclusively in the proximal ligand sphere like in the structurally closely-related complexes $[Ru(tbbpy)_3]^{2+}$ or $[(tbbpy)_2Ru(phen)]^{2+}$ (phen = 1,10-phenanthroline).[11, 12, 13] The corresponding dynamics is typically described quantitatively by two components (characteristic time-constants), namely the population of a long-lived state *via* intersystem crossing, vibrational cooling and inter-ligand hopping (k_0), which radiatively decays back to the ground state on the sub-μs timescale (k_1).[14, 11, 12, 13] Therefore, two rate constants are defined and added to the `lmfit` parameter object *via* `par.add('k0', value=1/2, vary=True)` and `par.add('k1', value=1/180000, vary=False)` (see section 4.1).

4.0.2 Working with *Jupyter* Notebooks

KiMoPack is written in `python` and can be installed by the common package managers `conda`[15] or `pypi`[16]. We recommend to use *Jupyter* notebooks for the analysis, allowing a suitable interface for working on the data using interactive plots and various parameters and functions. To open the notebook, you first need to install python and *Jupyter* notebook on your computer. The easiest way to do this, is to download and install the *Anaconda* package or to install only *Jupyter*. After installation, you need to install some extra packages, namely `qt` (for active plots) and `lmfit` (for fitting):

```
conda install -c anaconda qt
conda install -c conda-forge lmfit
```

Tutorials on working with *Jupyter* Notebooks can be found elsewhere, *e.g.*, the *Jupyter* website. Briefly, to use the program, open *Jupyter* Notebook, either directly from a terminal or from the *Anaconda* Navigator. This should open a new tab in your browser. The way the notebooks work is that you have cells with blocks of code. If you click on a cell and hit `Run` at the top (or Shift+Enter), it will execute the code in this cell. If you keep hitting `Run`, it will run the following cell, so to run the whole script, just keep hitting run, you do not have to select every cell manually.

Over the last decades a step-wise approach to transient data analysis has been established, through which the user is guided with the *Jupyter* worksheets that accompany **KiMoPack**. The `Jupyter` notebooks contain the typical parameters that are adjusted during a representative analysis session, so that new users can easily change (and comment) the values and experienced users have a customizeable and reproducible workflow. Typically, a new `Jupyter` notebook is created from the templates for each new analysis which then documents the procedures, parameters and results in a visible way, but also enables very fast data processing. If *e.g.* two similar data sets are to be analyzed, the time from importing the second file to the adjusted and saved results is despite being only a few seconds very reliable, provide meaningful results and can be used to compare the two datasets or create *e.g.* an experimental logfile. To learn their usage we recommend our tutorial notebooks that

contain two extensively commented analysis sessions. Typically a new Jupyter notebook is created from the templates for each new analysis which then documents the procedures, parameters and results in a visible way, but also enables very fast data processing.

4.1 Tutorial 01_KiMoPack_Fitting-1.ipynb

[1]:
```
import os,sys
import pandas as pd
import numpy as np
import matplotlib,lmfit
import matplotlib.pyplot as plt
try:
    import KiMoPack.plot_func as pf
except:
    print("General installation did not work, try to import from the same folder as␣
 ↪a workaround")
    import plot_func as pf
```

```
Plot_func version 6.2.8
was imported from path:  (path)
The current working folder is: (path)
```

4.1.1 Load Data

In the first step, the raw data must be passed to the TA object. Herein, it is demonstrated how to load a single measurement file. For learning how to load and average multiple scans see the KiMoPack_Rudppz-tutorial_ScanHandling.ipynb

In this example, the transient absorption data of a Ru-complex, namely $[(tbbpy)_2Ru(dppz)]^{2+}$, collected upon 400 nm excitation in three different solvents, namely dichloromethane (DCM), acetonitrile (ACN) and water (H_2O) is analysed. The tutorial is structured in such a way that only the data recorded in one solvent can be imported and analysed. To change the solvent, the solvent must be adjusted by the solvent parameter in the following cell and all subsequent steps must be repeated.

[2]:
```
solvent = 'ACN' #'DCM' or 'H2O'
filename = 'TA_Ru-dppz_400nm_'+str(solvent) # set name of the file to fit
# set path to file to fit
filepath = os.path.join(os.getcwd(), 'Data', 'Fitting-1')
```

The data is loaded by specifying the filename (string) and path of the TA-data file (string, path-variable).

[3]:
```
ta=pf.TA(filename=filename+'.SIA', # title of the measurement file
         path=filepath)             # path to measuremnt file
```

Hint: if the work with folders and filenames is cumbersome, by replacing the filename with "gui" a window pops up and a file can be selected with the mouse. »ta=pf.TA('gui')«

4.1.2 Background correction and filtering

- `Filter_data`: remove artificial data points |ΔOD| > 20
- `Background`: substract background before time_zero

[4]:
```
ta.Filter_data(value=20)       # remove artificial values
ta.Background(uplimit=-0.5)    # subtract background before time zero
```

4.1.3 Arrival time correction

Correct for different arrival times of different probe wavelength (sometimes called chirp) using `Man_chirp` or `Cor_Chirp` (checks for existing chirp data). The funtion opens an interactive 2D-contour plot of the TA data in a specified delay-time window. A desired colour map can be chosen to enable a good correction (see https://matplotlib.org/stable/tutorials/colors/colormaps.html).

- Firstly, the **intensity range** can be altered by clicking (left-click) on the scale on top of the plot. Once a suitable range is found it needs to be accepted on the lower left button.
- Secondly, user selects (left-click) a number of points along the **dispersion curve**, which are passed to a 4th order polynomial approximation by a middle-click.
- Thirdly, the point that is declared as **time zero** can be selected (right-click) and confirmed (accept-button).

In all of the selections a left click selects, a right click removes the last point and a middle click (sometimes abbreviated by clicking left and right together) finishes the selection. If no middle click exists, the process automatically ends after `max_points` (default: 40).

Try `ta.Man_Chirp(shown_window=[-2.3,1.8])` to follow the chirp correction on your own

[5]:
```
%matplotlib qt
# choose time-window used in the active plot
ta.Cor_Chirp(shown_window=[-2.3,1.8])
```

Note, "shown_window" is a special option chosen here because the arrival time correction needs to be performed over an extended range. Without this option -1ps to 1ps is the Default range.

4.1.4 Plot the standard corrected data

In this example the pre-processed data is visualized in three plots (as indicated in the titles), namely as kinetic traces (x: Δt, y: ΔAbsorbance), transient spectra (x: λ_{probe}, y: ΔAbsorbance) and 2D-contour plot (x: λ_{probe}, y: Δt, z: ΔAbsorbance). Several features can be sused to alter the appearance of those plots (see Documentation or type `ta.Plot?` in the notebook).

The parameters `rel_time` and `rel_wave` are used to pre-select interesting Δt and λ_{probe} values to show specific kinetic traces (`plotting=[1]`) or transient spectra (`plotting=[2]`) of the dataset. The `scattercut` argument takes a probe wavelength interval that is ignored (set to zero) in the plots, to suppress the plotting of scattered excitation light. Here the scatter region was found to be between 380 and 405 nm (excitation at 400 nm). The `time_width_percent` variable is set to 5%, meaning that the transient spectra are shown at the given delay time plus/minus 5% of that value (e.g. 0.522 ps means 0.5 to 0.55 ps). The respective range is indicated in the legend of the transient spectra. In all plots the unfitted data is plotted as dots, interpolated with lines (Savitzky-Golay).

```
[6]: %matplotlib qt
# certain delay times for TA spectra plot
ta.rel_time=[0.5,1.5,20,100,500]
# certain probe wavelengths for kinetic traces plot
ta.rel_wave=[350,440,520,600]
# ignored probe wavelength region (set to zero)
ta.scattercut=[380,405]
# number in percent defining a delay time region plotted in the TA spectra
ta.time_width_percent=5
ta.Plot_RAW(title='Kinetic traces at selected probe wavelengths', plotting=[1])
ta.Plot_RAW(title='TA spectra at selected delay-times', plotting=[2])
ta.Plot_RAW(title='2D-Plot', plotting=[0])
```

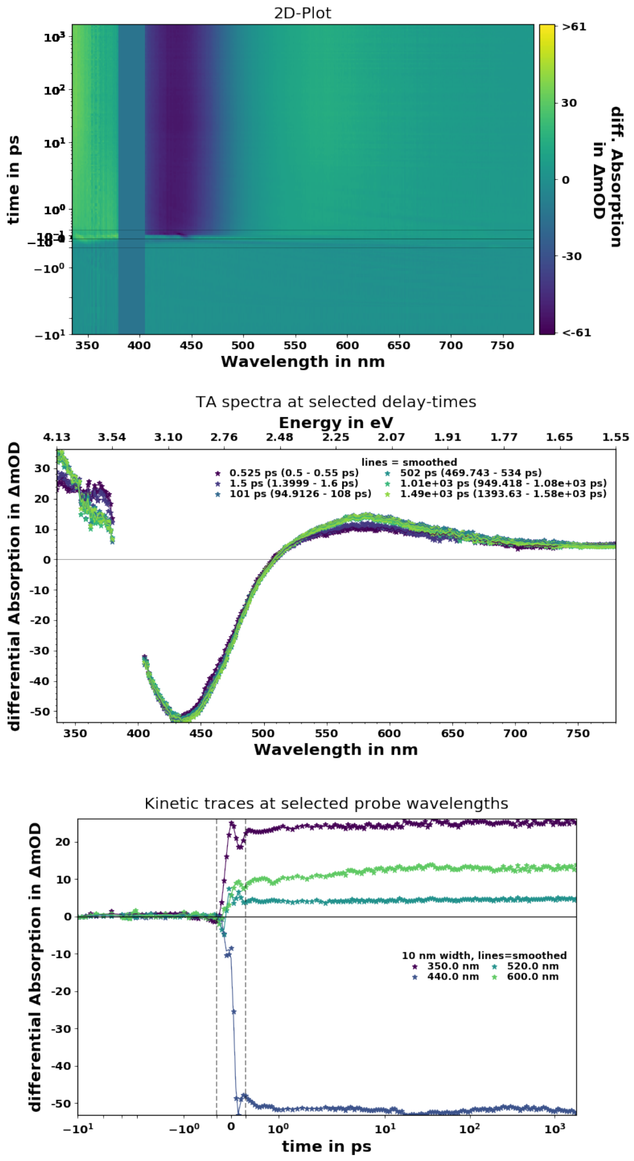

Figure S3: Plotting the shaped data (Raw plotting) as 2D matrix (top), spectra at selected times (middle) and kinetic traces for selected times (bottom)

4.1.5 Plot Singular Value Decomposition (SVD) Results

To estimate the number of processes that contribute to the overall spectral changes singular value decomposition can be used. Herein, the SVD component strength plot reveals that *circa* three components need to be used to describe the overall TA data.

[7]:
```
ta.Plot_RAW(title='SVD', plotting=[3], savetype='pdf')
```

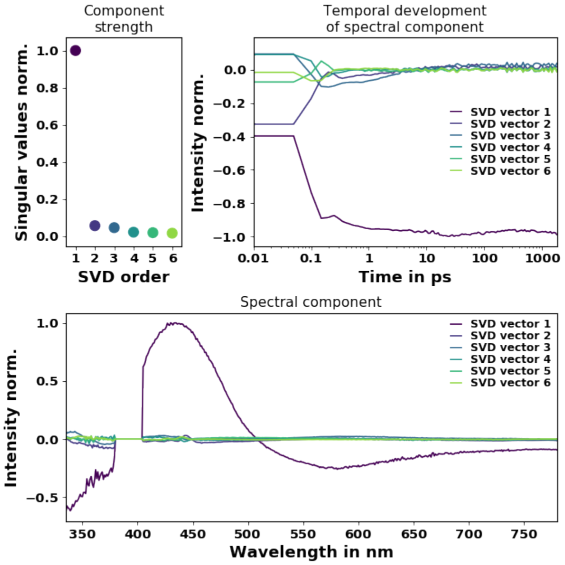

Figure S4: Plot of Single Value Decomposition showing the single value (component strength) top left, the temporal evolution of the vector in the top right and the spectrum of the main vectors in the bottom

4.1.6 Fitting of the Data

4.1.6.1 Define fitting parameters Upon photoexcitation of $[(tbbpy)_2Ru(dppz)]^{2+}$ (**Ru-dppz**) at 400 nm an ensemble of metal-to-ligand charge-transfer (MLCT) states localized in both ligand spheres, *i.e.*, $^1MLCT_{tbbpy}$ and $^1MLCT_{dppz}$ is populated. Extensive photophysical studies revealed that the subsequent excited state dynamics is determined by the polarity and hydrogen bond donor ability of the surrounding solvent molecules. It was found that long-lived emissive states are populated in polar aprotic solvents. However, this emission switches *off* when the molecules interact with water. This interesting property is based on a solvent sensitive excited state equilibrium between a non-emissive and an emissive state localized on the phenazine (phz) and phenanthroline (phen) moiety of the dppz ligand.

Parameter for Dichloromethane Several studies in dichloromethane solution reveal that primarily the *bright* phen-centered state ($^3MLCT_{phen}$) is populated by intersystem crossing, vibrational cooling and inter ligand hopping. This *bright* state radiatively decays within *circa* 180 ns. Hence, the photoinduced dynamics in dichloromethane is herein described by two characteristic time constants, namely `k0` and `k1`. The value for `k1` is fixed to 180 ns as obtained from nanosecond time-resolved studies. Additionally, a parameter for time zero (`t0`) and the pump-pulse width (`resolution`) are passed to the parameter object. For each parameter it can be specified whether it is optimized (`vary=True`) or kept constant (`vary=False`) in the fit.

```
[8]: ta.par=lmfit.Parameters()
# rate constants
ta.par.add('k0',value=1/2.0, min=1/10.0, max=1/0.25, vary=True)
ta.par.add('k1',value=180e3, vary=False)
# time-zero parameter fixed during fit
ta.par.add('t0',value=0.0, min=-0.1, max=0.1, vary=False)
# pump-pulse width parameter fixed during fit
ta.par.add('resolution', value=0.12, min=0.04, max=0.20, vary=False)
```

Parameter for Water The composition of the initially excited states is only minorly affected by the solvent. However, the phz-centered states are stabilized in aqueous environment due to a hydrogen bond interaction of the phz-nitrogen atoms with surrounding water molecules. Therefore, it was found that rapidly a phz-centered excited state is formed in water that non-radiatively decays back to the ground state within *circa* 2 ns. It is first a fast process with excited states with excess electron density on both, the phen and phz sphere of the dppz ligand are populated by intersystem crossing, vibrational cooling and inter ligand hopping. The dark phz-centered state is formed upon intraligand charge-transfer in a second step. Thus, also the excited state dynamics of **Ru-dppz** in H_2O can be described with three kinetic parameters (`k0`, `k1` and `k2`), that are added to the parameters object. In contrast to the initial guesses in ACN solution, `k3` is optimized during the fit and initially set to 2.1 ns.

```
[9]: ta.par=lmfit.Parameters()
# rate constants
ta.par.add('k0',value=1/0.5, vary=True)  # optimized during fit
ta.par.add('k1',value=1/100, vary=True)  # optimized during fit
ta.par.add('k2',value=1/1500, vary=True) # optimized during fit
# time-zero parameter fixed during fit
ta.par.add('t0',value=0.0, min=-0.5, max=0.5, vary=True)
# pump-pulse width parameterfixed during fit
ta.par.add('resolution', value=0.12, min=0.04, max=0.20, vary=False)
```

Parameter for Acetonitrile Extensive photophysical studies in acetonitrile revealed that due to the stabiization of the charge-transfer excited states, the *dark* phz-centered state is formed from the *bright* ^{3}MLCT$_{phen}$ state and decays back to the ground state on the sub-ns timescale. This formation of a long-lived long-lived ^{3}MLCT$_{phen}$ state is manifested, *i.e.*, in the spectral changes at 340 and 580 nm, which can be quantitatively described by two characteristic time-constants: the first one associated with intersystem crossing, vibrational cooling and interligand hopping and a second one attributed to the non-radiative decay of a subset of ^{3}MLCT states with excess electron density on the phenazine sphere of the dppz ligand (^{3}MLCT$_{phz}$), ultimately populating the long-lived ^{3}MLCT$_{phen}$ state. Hence, the three kinetic parameters `k0`, `k1` and `k2` are added to the the parameter object. The value for `k2` is fixed to 180 ns as obtained from nanosecond time-resolved studies.

```
[10]: ta.par=lmfit.Parameters()
# rate constants
ta.par.add('k0',value=1/2, min=1/10.0, vary=True)
ta.par.add('k1',value=1/150, min=1/200.0, vary=True)
ta.par.add('infinite') # or: ta.par.add('k2',value=180e3, vary=False)
# time-zero parameter fixed during fit
```

```
ta.par.add('t0',value=0.0, min=-0.1, max=0.1, vary=True)
# pump-pulse width parameter fixed during fit
ta.par.add('resolution', value=0.12, min=0.04, max=0.20, vary=False)
```

4.1.6.2 Kinetic Modeling (Parallel Model) In the parallel model a number of initially photoexcited states (herein N=3) are taken to decay independently from each other following first order kinetics. Thus the concentration profile for each component (C_{ac}) is decribed by an exponential decay ($\exp(-k_c \cdot \Delta t_a)$). If the width of the instrument-response function is negligible, the parallel model for the TA signals reads

$$\Delta A_{ab} = \sum_{c=1}^{N} C_{ab} \cdot S_{cb} \qquad \text{with } a \in \{\Delta t_1, \Delta t_1, \ldots, \Delta t_m\}, b \in \{\lambda_1, \lambda_2, \ldots, \lambda_n\}$$

The S_{cb} matrix represents the amplitudes of the exponential decays at each probe wavelength, namely the decay associated spectra. Negative contributions describe the build-up and positive contributions the decay of ΔA_{ab} signals.

Example: Parallel Model

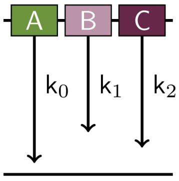

$$\frac{d[A]}{dt} = -k_0 \cdot [A]$$

$$\frac{d[B]}{dt} = -k_1 \cdot [B]$$

$$\frac{d[C]}{dt} = -k_2 \cdot [C]$$

```
ta.mod='exponential'
par=lmfit.Parameters()
par.add('k0',value=1/2,vary=True)
par.add('k1',value=1/150,vary=True)
par.add('k2',value=1/100000,vary=False)
```

Figure S5: Schematic sketch of the parallel model

```
[11]: # model selection (independent parallel decay)
ta.mod='exponential'
ta.timelimits=[0.3,2000]          # set maximum and minimum time for fit
# ta.ignore_time_region=[-0.2,0.3] # alternative to blind out the region around time=0
                                   # to avoid the artifacts but allow the use of pre-zero data
ta.Fit_Global()                   # global lifetime analysis (kinetic modeling)
```

```
we use adaptive mode for nelder

Fit Results:
Model Used: exponential

The minimum error is:1.91656427e-02
The minimum R2-value is:9.99222331e-01

In Rates

                  value   init_value   vary   min  max  expr
```

```
k0              0.270371            2   True     0  inf  None
k1             0.0105779         0.01   True     0  inf  None
k2            2.4758e-13  0.000666667   True     0  inf  None
t0           -0.00202596            0   True  -0.5  0.5  None
resolution          0.12         0.12  False  0.04  0.2  None
```

```
The rates converted to times with unit ps
```

```
                  value init_value   vary   min  max  expr
k0              3.69862        0.5   True     0  inf  None
k1              94.5365        100   True     0  inf  None
k2           4.0391e+12       1500   True     0  inf  None
t0          -0.00202596          0   True  -0.5  0.5  None
resolution         0.12       0.12  False  0.04  0.2  None
```

4.1.7 Plot Fit Results

[12]:
```
plt.close('all')   # as many plots will be generated this is a good habit.
ta.intensity_range=[-70e-3,30e-3]
# We choose a slightly different intensity range to make the dynamics visible
ta.Plot_fit_output(title='2D-Plots', plotting=[4])
```

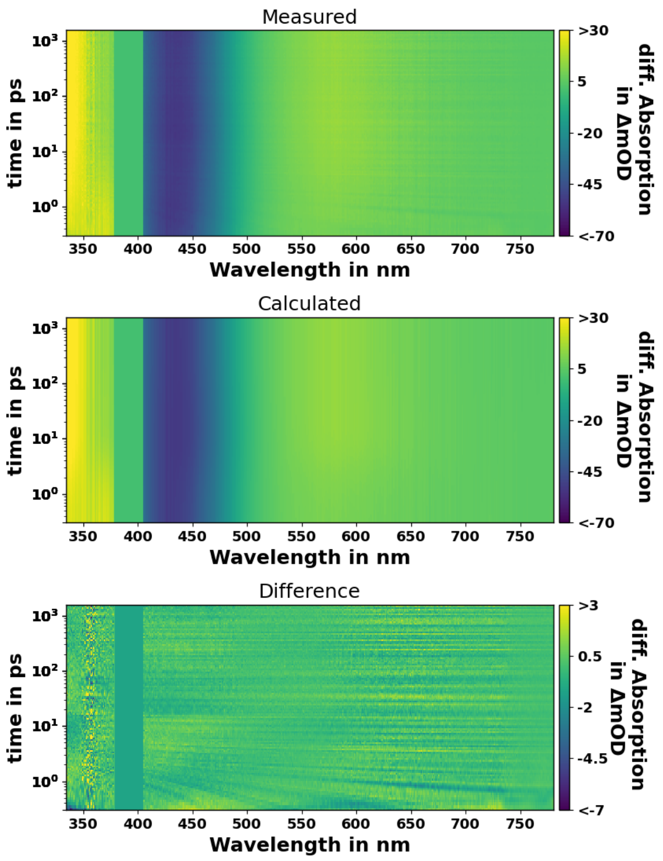

Figure S6: Plotting of the Measured (top), Modelled (middle) and difference matrix (bottom). Note the different intensity scales.

```
[13]: ta.Plot_fit_output(title='TA spectra', plotting=[3])
      ta.Plot_fit_output(title='kinetic traces', plotting=[2])
      ta.Plot_fit_output(title='summed TA signals', plotting=[1])
```

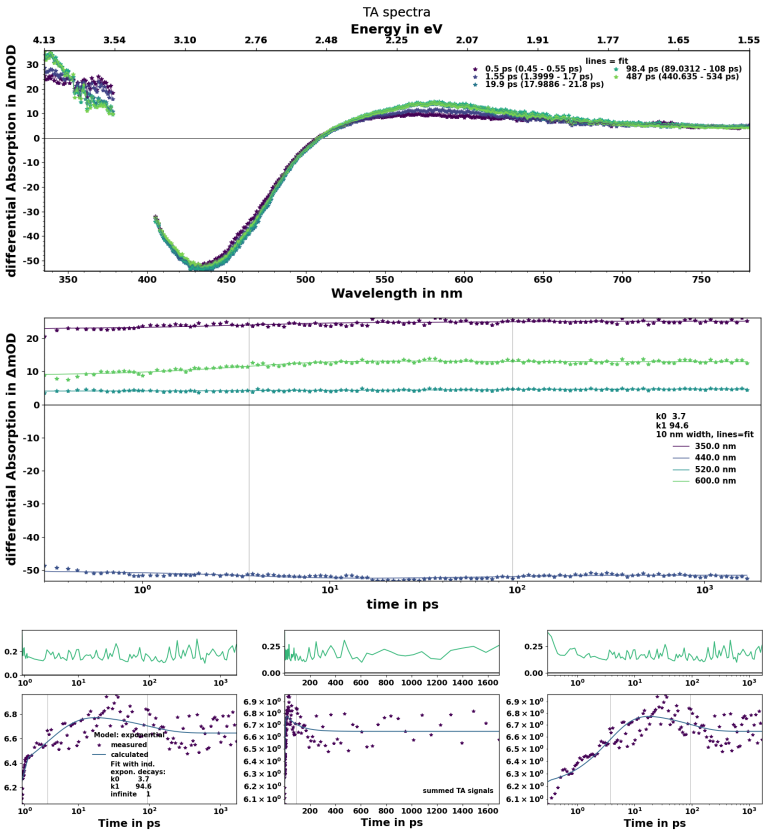

Figure S7: Plotting the fitted measured and fitted spectra (top), kinetics of selected wavelength (middle) and all wavelength summed (bottom). The selection of wavelength and time-points to plot is using the same parameter as for the RAW plotting above.

[14]:

```
ta.Plot_fit_output(title='Decay Associated Spectra', plotting=[0])
ta.Plot_fit_output(title='concentration profiles', plotting=[5])
```

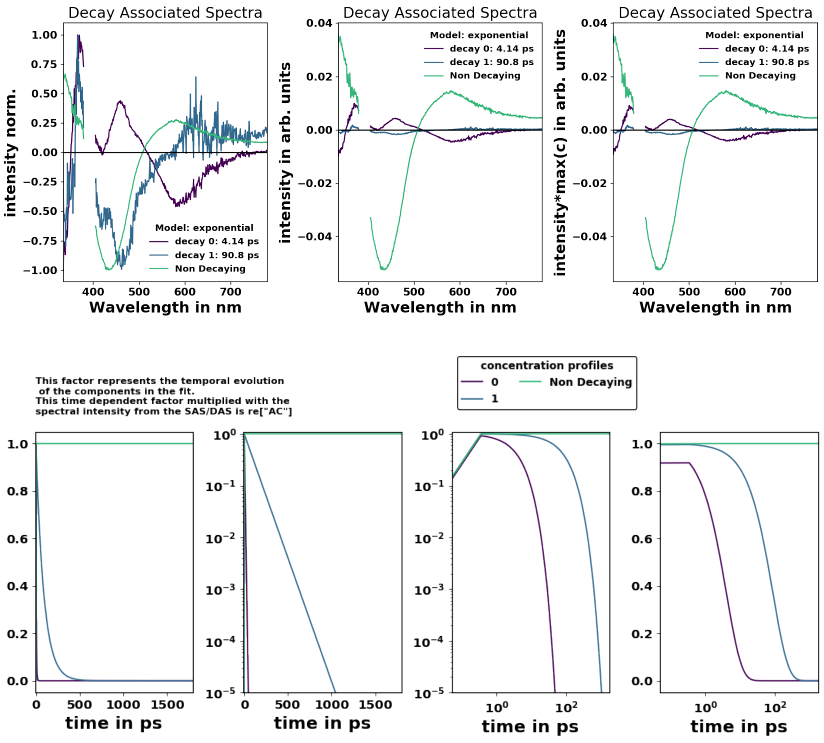

Figure S8: Plotting the extracted DAS/SAS (top) and the temporal evolution of the species/concentrations (bottom)

4.1.8 Save Results

The whole project, including *e.g.* the fit parameters, settings for the plots and fit results can be saved by the `Save_project` function. Thereby a *hdf5* file is written to the specified output folder. The pre-processed data (`save_RAW=True`) as well as the fitted data and fit results (`save_Fit=True`) can be saved as ascii files to a specified folder by `Save_data`. Details of the exported files are given in Section Data Export

Additionally the graphs can be saved by the `Save_Plots` function. Thereby, temporarily the `save_plots_to_folderoption` is set to true whereby the pre-processed (*e.g.* *_RAW_SPEK.pdf, _RAW_SEL.pdf) *and fitted* (e.g.* _DAC.pdf, _concentrations.pdf*) data are replotted.

If just the standard filenames should be used then an empty call to the saving functions is sufficient.

[15]:

```
ta.Save_project()
ta.Save_data()
ta.Save_Powepoint()
```

In the example, the files are saved to the folder of the raw data in a subfolder named 'results_solvent'. Additionally some other options are chosen to select only specific files.

```
[16]: ta.Save_project(filename=filename+'_paral',              # set save name
                     path='results_'+str(solvent))             # set name of save folder

      ta.Save_data(save_RAW=False,                     # do not save the pre-processed data
                   save_Fit=True,                              # save the fitted data
                   filename=filename+'_paral',                 # set save name
                   path='results_'+str(solvent))               # set name of save folder

      #ta.Save_Plots(path='results_'+str(solvent),              # set name of save folder
      #               title='Ru-dppz, 400nm, '+str(solvent),    # set plot titles
      #               filename=filename+'_paral',              # set save name
      #               savetype='pdf',                          # set save type
      #               cmap=pf.cm.PiYG)                         # define colormap

      ta.Save_Powerpoint(save_RAW=False,                   # do not save pre-processed data
                         save_Fit=True,                        # save fitted data
                         filename=filename+'_paral',           # set save name
                         path='result_summary',                # set name of save folder
                         savetype='pdf')                 # set savetype (pdf, svg or pptx)
```

```
The project was saved to (path to files)
```

4.1.8.1 Kinetic Modeling (Sequential Model) In the sequential model initially one component is excited and irreversibly decays forming a second component. Subsequently this component irreversibly decays populating a second component. this consecutive decay repeats over multiple states (in this example three states). Like in the parallel model, the TA signals are described by the product of a concentration matrix of three species and their respective spectral weights. The latter are called species associated spectra. Each j-th SAS is a combination of the j-th and the following decay associated spectrum. Thus, the final species associated spectrum is direct proportional to the final decay associated spectrum.

Example: Sequential Model

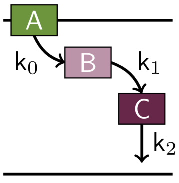

$$\frac{d[A]}{dt} = -k_0 \cdot [A]$$

$$\frac{d[B]}{dt} = k_0 \cdot [A] - k_1 \cdot [B]$$

$$\frac{d[C]}{dt} = k_1 \cdot [B] - k_2 \cdot [C]$$

```
ta.mod='consecutive'
par=lmfit.Parameters()
par.add('k0',value=1/2,vary=True)
par.add('k1',value=1/150,vary=True)
par.add('k2',value=1/100000,vary=False)
```

Figure S9: Schematic sketch of the sequential model

To fit the data within own-defined models, *i.e.*, other models than,

```
'exponential', 'consecutive', 'full_consecutive'
```

see the `02_KiMoPack_Fitting-2.ipynb` tutorial.

[17]:
```
# model selection (independent parallel decay)
ta.mod='consecutive'

# global lifetime analysis (kinetic modelling)
ta.Fit_Global()

# plot results (summed kinetics)
ta.Plot_fit_output(plotting=[1])
```

```
Fit Results:
Model Used: consecutive

The minimum error is:1.92131332e-02
The minimum R2-value is:9.99220404e-01

In Rates

                   value  init_value   vary    min  max  expr
k0               0.27035         0.5   True    0.1  inf  None
k1             0.0105748  0.00666667   True  0.005  inf  None
infinite               1           1  False   -inf  inf  None
t0           0.000126229           0   True   -0.1  0.1  None
resolution          0.12        0.12  False   0.04  0.2  None

The rates converted to times with unit ps

                   value init_value   vary   min  max  expr
k0               3.69891          2   True     0   10  None
k1               94.5642        150   True     0  200  None
infinite               1          1  False  -inf  inf  None
t0           0.000126229          0   True  -0.1  0.1  None
resolution          0.12       0.12  False  0.04  0.2  None
```

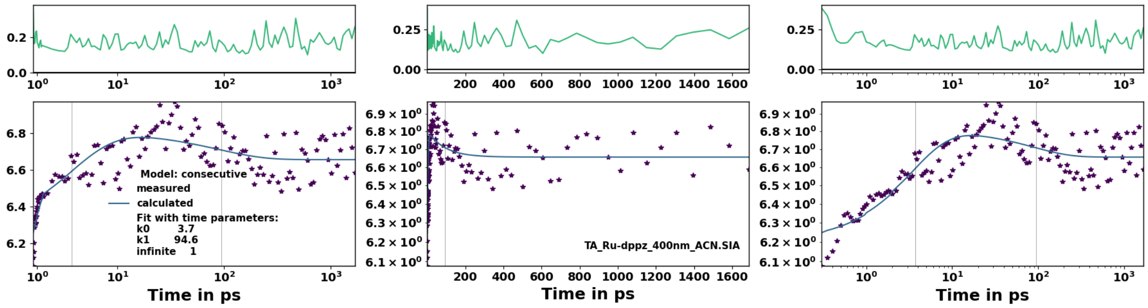

Figure S10: Plotting the integrated spectra intensities of this new model

Save Results

```
[22]: ta.Save_project(filename=filename+'_seq',      # set save name
                      path='results_'+str(solvent))  # set name of save folder
```

```
The project was saved to (path)
```

4.2 Tutorial 02_KiMoPack_Fitting-2.ipynb

[1]:
```
import os,sys
import pandas as pd
import numpy as np
import matplotlib,lmfit
import matplotlib.pyplot as plt
try:
    import KiMoPack.plot_func as pf
except:
    print("General installation did not work, import from the same folder as a␣
 ↪workaround")
    import plot_func as pf
```

```
Plot_func version 6.20
was imported from path:
 (path)
The current working folder is:
 (path)
```

4.2.1 Import data

[2]:
```
solvent = 'ACN' #'DCM' or 'H2O'
filename = 'TA_Ru-dppz_400nm_'+str(solvent) # set name of the file to fit
filepath = os.path.join(os.getcwd(), 'Data', 'Fitting-2')  # set path
ta=pf.TA(filename=filename+'.SIA', # title of the measurement file
         path=filepath) # path to measuremnt file
#Alternative:
# ta=pf.TA('gui')   #and navigate to the corresponding file
```

4.2.2 Standard corrections

[3]:
```
%matplotlib qt
ta.Filter_data(value=20) # remove artificial values
ta.Background(uplimit=-0.5) # subtract background before time zero
ta.Cor_Chirp(shown_window=[-2.3,1.8]) # choose time-window for active plot
```

4.2.3 Plot pre-processed data

In this example the pre-processed data is visualized in three plots (as indicated in the titles),

1. as kinetic traces (x: Δt, y: ΔAbsorbance)
2. transient spectra (x: λ_{probe}, y: ΔAbsorbance)
3. 2D-contour plot (x: λ_{probe}, y: Δt, z: ΔAbsorbance).

Several features can be used to alter the appearance of those plots (see Documentation or type `ta.Plot?` in the notebook).

- The parameters `rel_time` and `rel_wave` are used to pre-select interesting Δt and λ_{probe} values to show specific kinetic traces (`plotting=1`) or transient spectra (`plotting=2`) of the dataset.

- The parameters timelimits, bordercut and intensity_range are specified to control the displayed region, by specifying upper and lower limits of delay times, probe wavelengths and TA signal intensities, respectively.
- The scale of the TA signals can be changed to a logarithmic scaling using log_scale=True.

- The scattercut argument takes a probe wavelength interval that is ignored (set to zero) in the plots, to suppress the plotting of scattered excitation light. Here the scatter region was found to be between 380 and 405 nm (excitation at 400 nm).
- The time_width_percent variable is set to 5%, meaning that the transient spectra are shown at the given delay time plus/minus 5% of that value (e.g. 0.522 ps means 0.5 to 0.55 ps). The respective range is indicated in the legend of the transient spectra. In all plots the unfitted data is plotted as dots, interpolated with lines (Savitzky-Golay).

```
[4]: %matplotlib qt
ta.rel_time=[0.5,1.5,20,100,1000]  # delay times for TA spectra plot
ta.rel_wave=[350,440,520,600]      # probe wavelengths for kinetics plot
ta.timelimits=[-1,1400]            # plotted delay time range
ta.bordercut=[320,770]             # plotted probe wavelength range
ta.intensity_range=[-55e-3,55e-3]  # plotted intensity range
ta.scattercut=[378,407]            # ignored probe wavelength region
ta.time_width_percent=5 # number in percent defining a delay time region
ta.Plot_RAW(title='Kinetic traces at selected probe wavelengths', plotting=1)
ta.Plot_RAW(title='TA spectra at selected delay-times', plotting=2)
ta.Plot_RAW(title='2D-Plot', plotting=0)
```

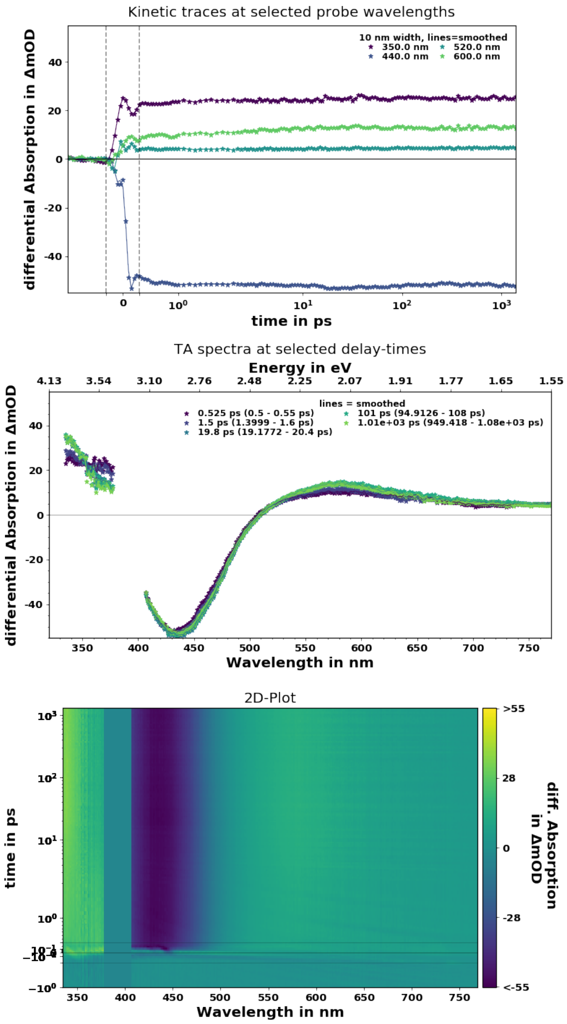

Figure S11: Separate plotting of the as-measured selected Kinetic traces (top), selected spectra (middle) and the as-measured matrix (bottom)

4.2.4 Fitting of the data

4.2.4.1 Global analysis - parallel Model Upon photoexcitation of $[(tbbpy)_2Ru(dppz)]^{2+}$ (**Ru-dppz**) at 400 nm an ensemble of metal-to-ligand charge-transfer (MLCT) states localized in both ligand spheres, *i.e.*, $^1MLCT_{tbbpy}$ and $^1MLCT_{dppz}$ is populated. Extensive photophysical studies revealed that the subsequent excited state dynamics is determined by the polarity and hydrogen bond donor ability of the surrounding solvent molecules. It was found that long-lived emissive states are populated in polar aprotic solvents. However, this emission switches *off* when the molecules interact with water. This interesting property is based on a solvent sensitive excited state equilibrium between a non-emissive and an emissive state localized on the phenazine (phz) and phenanthroline (phen) moiety of the dppz ligand.

Extensive photophysical studies in acetonitrile revealed that due to the stabiization of the charge-transfer excited states, the *dark* phz-centered state is formed from the *bright* $^3MLCT_{phen}$ state and decays back to the ground state on the sub-ns timescale. This formation of a long-lived long-lived $^3MLCT_{phen}$ state is manifested, *i.e.*, in the spectral changes at 340 and 580 nm, which can be quantitatively described by two characteristic time-constants: the first one associated with intersystem crossing, vibrational cooling and interligand hopping and a second one attributed to the non-radiative decay of a subset of ^3MLCT states with excess electron density on the phenazine sphere of the dppz ligand ($^3MLCT_{phz}$), ultimately populating the long-lived $^3MLCT_{phen}$ state. Hence, the three kinetic parameters `k0`, `k1` and `k2` are added to the the parameter object. The value for `k2` is fixed to 180 ns as obtained from nanosecond time-resolved studies.

Example: Parallel Model

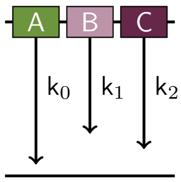

$$\frac{d[A]}{dt} = -k_0 \cdot [A]$$

$$\frac{d[B]}{dt} = -k_1 \cdot [B]$$

$$\frac{d[C]}{dt} = -k_2 \cdot [C]$$

```
ta.mod='exponential'
par=lmfit.Parameters()
par.add('k0',value=1/2,vary=True)
par.add('k1',value=1/150,vary=True)
par.add('k2',value=1/100000,vary=False)
```

Figure S12: Schematic sketch of the parallel model

[5]:

```
# Define fit parameters
ta.par=lmfit.Parameters()
# rate constants
ta.par.add('k0',value=1/2, min=1/10.0, max=1/0.25, vary=True)
ta.par.add('k1',value=1/150, min=1/200.0, max=1/10.0, vary=True)
ta.par.add('k2',value=1/100000, vary=False)
# time-zero parameter fixed during fit
ta.par.add('t0',value=0.0, min=-0.1, max=0.1, vary=False)
# pump-pulse width parameter fixed during fit
ta.par.add('resolution', value=0.12, min=0.04, max=0.20, vary=False)
# Select a in-build model (here: independent parallel decay)
ta.mod='exponential'
# set delay-time range for fit
ta.timelimits=[0.35,2000]
# global lifetime analysis (kinetic modeling)
```

```
ta.Fit_Global()
```

```
Fit Results:
Model Used: exponential
The minimum error is:2.69778430e-02
The minimum R2-value is:9.98959269e-01
In Rates
                value  init_value   vary    min  max  expr
k0           0.268506         0.5   True    0.1    4  None
k1          0.0175803  0.00666667   True  0.005  0.1  None
k2              1e-05       1e-05  False      0  inf  None
t0                  0           0  False   -0.1  0.1  None
resolution       0.12        0.12  False   0.04  0.2  None
The rates converted to times with unit ps
              value init_value   vary   min  max  expr
k0          3.72432          2   True  0.25   10  None
k1          56.8818        150   True    10  200  None
k2           100000     100000  False     0  inf  None
t0                0          0  False  -0.1  0.1  None
resolution     0.12       0.12  False  0.04  0.2  None
```

4.2.5 Repeat the global analysis to estimate the errors

[6]:
```
# fit-error estimation in a confidence interval of 95%
ta.Fit_Global(confidence_level=0.95)
```

```
Trying to find k0, lower confidence limit
Trying to find k0, upper confidence limit
Trying to find k1, lower confidence limit
Trying to find k1, upper confidence limit
it took 169 optimisations to get the confidence
Fit Results:
Model Used: exponential
The minimum error is:2.69778430e-02
The minimum R2-value is:9.98959269e-01
In Rates with confidence interval to level of 95.0
 value lower_limit upper_limit  init_value   vary    min  max expr
k0    0.268506    0.157326    0.497447         0.5   True    0.1    4  None
k1   0.0175803  0.00412181    0.111088  0.00666667   True  0.005  0.1  None
k2       1e-05        None        None       1e-05  False      0  inf  None
t0           0        None        None           0  False   -0.1  0.1  None
resolution 0.12        None        None        0.12  False   0.04  0.2  None
The rates converted to times with unit ps with confidence interval to
level of 95.0
      value lower_limit upper_limit init_value   vary   min  max  expr
k0      3.72432     2.01026     6.35622          2   True  0.25   10  None
k1      56.8818     9.00186     242.612        150   True    10  200  None
k2      100000        None        None     100000  False     0  inf  None
t0      0        None        None          0  False  -0.1  0.1  None
```

```
resolution   0.12        None        None       0.12  False  0.04  0.2  None
```

4.2.6 Plotting of error Analysis (advanced handling)

Plotting the results of the error analysis is challenging and due to the potential large amount of combinations not possible to perform automatic. However here is an example on a single parameter

[7]:
```
ta_listen=[ta.Copy(),ta.Copy()] #create a list for comparision
#the Filename can be manipulated to use the automatic naming
ta_listen[0].filename="upper confidence limit"
ta_listen[1].filename="lower confidence limit"
for i in range(2):
    #short name for the calculated results for reduced writing
    par=ta.re['fit_results_rates'].copy()
    if i == 0:
        #overwrite the value with the limits
        par.loc['k0','value']=par.loc['k0','upper_limit']
    else:
        par.loc['k0','value']=par.loc['k0','lower_limit']
    # Write the fit results as input parameter
    ta_listen[i].par=pf.pardf_to_par(par)
    for key in ta_listen[i].par.keys():
        # Lock the parameter so that only the spectra are calculated
        ta_listen[i].par[key].vary=False
    # Run the global fit to calculate the new spectra
    ta_listen[i].Fit_Global()
```

```
ATTENTION: we have not optimized anything but just returned the parameters
Fit Results:
Model Used: exponential
The minimum error is:2.78784275e-02
The minimum R2-value is:9.98924527e-01
In Rates
                value  init_value   vary    min  max  expr
k0           0.497447    0.497447  False    0.1    4  None
k1          0.0175803   0.0175803  False  0.005  0.1  None
k2              1e-05       1e-05  False      0  inf  None
t0                  0           0  False   -0.1  0.1  None
resolution       0.12        0.12  False   0.04  0.2  None
The rates converted to times with unit ps
              value init_value   vary   min  max  expr
k0          2.01026    2.01026  False  0.25   10  None
k1          56.8818    56.8818  False    10  200  None
k2           100000     100000  False     0  inf  None
t0                0          0  False  -0.1  0.1  None
resolution     0.12       0.12  False  0.04  0.2  None
ATTENTION: we have not optimized anything but just returned the parameters
Fit Results:
Model Used: exponential
```

```
The minimum error is:2.76709800e-02
The minimum R2-value is:9.98932530e-01
In Rates
                value  init_value   vary    min   max  expr
k0           0.157326    0.157326  False    0.1     4  None
k1          0.0175803   0.0175803  False  0.005   0.1  None
k2              1e-05       1e-05  False      0   inf  None
t0                  0           0  False   -0.1   0.1  None
resolution       0.12        0.12  False   0.04   0.2  None
The rates converted to times with unit ps
              value  init_value   vary    min   max  expr
k0          6.35622     6.35622  False   0.25    10  None
k1          56.8818     56.8818  False     10   200  None
k2           100000      100000  False      0   inf  None
t0                0           0  False   -0.1   0.1  None
resolution     0.12        0.12  False   0.04   0.2  None
```

4.2.6.1 Plotting and Shaping of the plot The plot could be re-shaped using the plotting GUI. Here we need to use a trick to achieve this automatically. Firstly, we get a handle to the drawn axis, so that we can directly manipulate the plot. Secondly, we set the ylimit of the plot.

[15]:
```
%matplotlib qt
ta.Compare_at_wave(fitted=True,
                   other=ta_listen,
                   rel_wave=[450,590],
                   width=50,
                   linewidth=3)
ax=plt.gca()              # get a handle to the drawn axis
ax.set_ylim(-50e-3,20e-3)  # set the ylimit of the plot
```

[15]:
```
(-0.05, 0.02)
```

4.2.7 Plotting of error Analysis (advanced handling)

Plotting of the results of the error analysis is challenging and due to the potential large amount of combinations not possible to perform automatic. However here is an example on a single parameter

[7]:
```
ta_listen=[ta.Copy(),ta.Copy()] #create a list for comparision
#the Filename can be manipulated to use the automatic naming
ta_listen[0].filename="upper confidence limit"
ta_listen[1].filename="lower confidence limit"

for i in range(2):
    #short name for the calculated results for reduced writing
    par=ta.re['fit_results_rates'].copy()
    if i == 0:
        #overwrite the value with the limits
        par.loc['k0','value']=par.loc['k0','upper_limit']
    else:
```

```
        par.loc['k0','value']=par.loc['k0','lower_limit']
    # Write the fit results as input parameter
    ta_listen[i].par=pf.pardf_to_par(par)
    for key in ta_listen[i].par.keys():
        # Lock the parameter so that only the spectra are calculated
        ta_listen[i].par[key].vary=False
    # Run the global fit to calculate the new spectra
    ta_listen[i].Fit_Global()
```

```
ATTENTION: we have not optimized anything but just returned the parameters

Fit Results:
Model Used: exponential

The minimum error is:1.92539718e-02
The minimum R2-value is:9.99202429e-01

In Rates

                value init_value   vary    min  max  expr
k0            0.54532    0.54532  False    0.1    4  None
k1          0.0142909  0.0142909  False  0.005  0.1  None
k2              1e-05      1e-05  False      0  inf  None
t0                  0          0  False   -0.1  0.1  None
resolution       0.12       0.12  False   0.04  0.2  None

The rates converted to times with unit ps

              value init_value   vary   min  max  expr
k0          1.83378    1.83378  False  0.25   10  None
k1          69.9745    69.9745  False    10  200  None
k2           100000     100000  False     0  inf  None
t0                0          0  False  -0.1  0.1  None
resolution     0.12       0.12  False  0.04  0.2  None
ATTENTION: we have not optimized anything but just returned the parameters

Fit Results:
Model Used: exponential

The minimum error is:1.84388291e-02
The minimum R2-value is:9.99236195e-01

In Rates

                value init_value   vary    min  max  expr
k0           0.156036   0.156036  False    0.1    4  None
k1          0.0142909  0.0142909  False  0.005  0.1  None
k2              1e-05      1e-05  False      0  inf  None
t0                  0          0  False   -0.1  0.1  None
```

```
resolution       0.12        0.12  False   0.04  0.2  None

The rates converted to times with unit ps

              value init_value   vary   min  max  expr
k0          6.40878    6.40878  False  0.25   10  None
k1          69.9745    69.9745  False    10  200  None
k2           100000     100000  False     0  inf  None
t0                0          0  False  -0.1  0.1  None
resolution     0.12       0.12  False  0.04  0.2  None
```

4.2.8 Shaping of the plot

The plot could be re-shaped using the plotting GUI. Here we need to use a trick to achieve this automatically. Firstly, we get a handle to the drawn axis, so that we can directly manipulate the plot. Secondly, we set the ylimit of the plot.

[13]:
```
ta.Compare_at_wave(fitted=True,
                   other=ta_listen,
                   rel_wave=[450,590],
                   width=50,
                   linewidth=3)

ax=plt.gca()               # get a handle to the drawn axis
ax.set_ylim(-50e-3,20e-3)  # set the ylimit of the plot
```

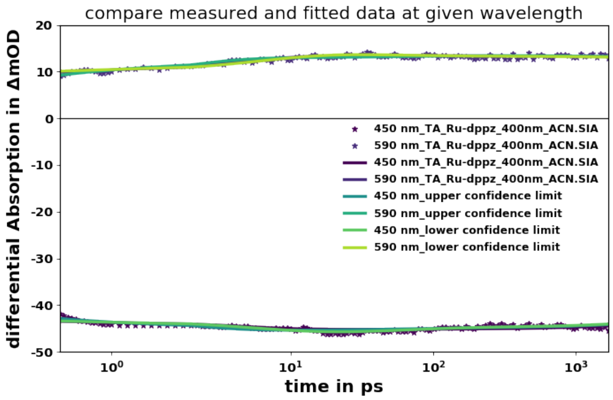

Figure S13: Comparison plot between the kinetics at two selected wavelength and how the kinetics would be at the confidence limits of the fit.

4.2.9 Target analysis – propose a model

Several studies in acetonitrile reveal that the *dark* phz-centered excited state is populated from the initially excited ^{3}MLCT states. Ultimately, a *bright* ^{3}MLCT$_{phen}$ is formed from the tbbpy, phen and phz centered states. Herein, it is shown how to define an own model function based on such *a priori* knowledge.

4.2.10 Define own model function

Based on the literature findings a model is proposed, where initially proximal MLCT (tbbpy and phen, A) and distal MLCT states (phz, B) are populated. Subsequently those states decay forming the *bright* MLCT_{phen} state (C). This in turn decays back to the ground state (C′). The respective kinetic rate constants can be written as

- $\frac{d[A]}{dt} = -k_0 \cdot [A]$
- $\frac{d[B]}{dt} = -k_0 \cdot [B]$
- $\frac{d[C]}{dt} = k_0 \cdot ([A] + [B]) - k_1 \cdot [C]$,

with brackets indicating the concentration of A, B and C. Those rate equations are defined in the python function, like:

```
# state: A, d[A]/dt
dc[0] = -pardf['k0']*dt*c_temp[0] + g[i]*dt
# state: B, d[B]/dt
dc[1] = -pardf['k0']*dt*c_temp[1] + g[i]*dt
# state: C, d[C]/dt
dc[2] = pardf['k0']*dt*c_temp[0] + pardf['k0']*dt*c_temp[1] - pardf['k1']*dt*c_temp[2]
```

Example: Complex Model

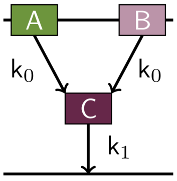

$$\frac{d[A]}{dt} = -k_0 \cdot [A]$$

$$\frac{d[B]}{dt} = -k_0 \cdot [B]$$

$$\frac{d[C]}{dt} = k_0 \cdot ([A] + [B]) - k_1 \cdot [C]$$

```
def Rudppz(times,pardf):
    ...
mod=Rudppz
par=lmfit.Parameters()
par.add('k0',value=1/4,vary=True)
par.add('k1',value=1/100000)
```

Figure S14: Schematic sketch of the user-defined model

[9]:
```
FWHM=2.35482
def gauss(t,sigma=0.1,mu=0):
    y=np.exp(-0.5*((t-mu)**2)/sigma**2)
    y/=sigma*np.sqrt(2*np.pi)
    return y
def Rudppz(times,pardf):
        '''
        Define a model where initially A and B are populated and decay
        forming C. Subsequently, C decays back to the ground-state
        args:
                pardf: pandas.DataFrame
                times: vector (type:list)
        returns:
                c: DataFrame with the times as index and in the columns as
                an expression of the relative concentrations of A, B and
                C (type: dictionary)
```

```
        '''
        # create an empty concentration matrix
        c=np.zeros((len(times),3),dtype='float')
        # create IRF
        g=gauss(times,sigma=pardf['resolution']/FWHM,mu=pardf['t0'])
        # defining step-size (taken between the main time_points)
        sub_steps=10
        for i in range(1,len(times)):
                # initial change for each concentration (3 refers to the number of␣
↪states)
                dc=np.zeros((3,1),dtype='float')
                # adaption of the time-intervals to the sub_steps
                dt=(times[i]-times[i-1])/(sub_steps)
                # create a temporary concentration matrix
                c_temp=c[i-1,:]
                for j in range(int(sub_steps)):
                        # state: A, d[A]/dt
                        dc[0] = -pardf['k0']*dt*c_temp[0] + g[i]*dt
                        # state: B, d[B]/dt
                        dc[1] = -pardf['k0']*dt*c_temp[1] + g[i]*dt
                        # state: C, d[C]/dt
                        dc[2] = pardf['k0']*dt*c_temp[0] + pardf['k0']*dt*c_temp[1]␣
↪- pardf['k1']*dt*c_temp[2]
                        for b in range(c.shape[1]):
                                #check that all concentrations are > 0
                                c_temp[b] =np.nanmax([(c_temp[b]+dc[b]),0.])
                # store temporary concentrations into the main matrix
                c[i,:] =c_temp
        c=pd.DataFrame(c,index=times)
        c.index.name='time' # name the delay-times
        c.columns=['A','B','C'] # name the species
        return c
```

4.2.11 Define fitting parameters

[10]:
```
ta.par=lmfit.Parameters()
# rate constants
ta.par.add('k0',value=1/2.0, min=1/10.0, max=1/0.1, vary=True)
ta.par.add('k1',value=1/100000, vary=False)
# time-zero parameter fixed during fit
ta.par.add('t0',value=0.0, min=-0.1, max=0.1, vary=False)
# pump-pulse width parameter fixed during fit
ta.par.add('resolution', value=0.07, min=0.04, max=0.20, vary=False)
```

4.2.12 Fitting of the Data - Kinetic Modeling (Ru-dppz Model)

[11]:
```
ta.mod=Rudppz                 # model selection (own model)
ta.timelimits=[0.35,2000]     # set delay-time range for fit
ta.log_fit=False              # fitting on linear time scale
ta.Fit_Global()               # pass parameter object (par) to global fit
```

```
0.03287224701630877
0.03104424588743773
0.029708659958358023
0.029080954685478902
0.030363339027524767
0.02932526602258752
0.029056994294598425
0.029056992333062033
0.029056992119748992
Fit Results:
Model Used: External function
The minimum error is:2.90569921e-02
The minimum R2-value is:9.98879062e-01
In Rates
               value init_value   vary   min  max  expr
k0          0.215485        0.5   True   0.1   10  None
k1             1e-05      1e-05  False     0  inf  None
t0                 0          0  False  -0.1  0.1  None
resolution      0.07       0.07  False  0.04  0.2  None
The rates converted to times with unit ps
             value init_value   vary   min  max  expr
k0          4.6407          2   True   0.1   10  None
k1          100000     100000  False     0  inf  None
t0               0          0  False  -0.1  0.1  None
resolution    0.07       0.07  False  0.04  0.2  None
```

Plot the fit results

[16]:
```
plt.close('all')
%matplotlib qt
ta.Plot_fit_output(title='2D-Plots', plotting=4)
```

[13]:
```
ta.Plot_fit_output(title='summed TA signals', plotting=1)
ta.Plot_fit_output(title='Decay Associated Spectra', plotting=0)
#ta.Plot_fit_output(title='concentration profiles', plotting=5)
#or: ta.re['c'].plot()
```

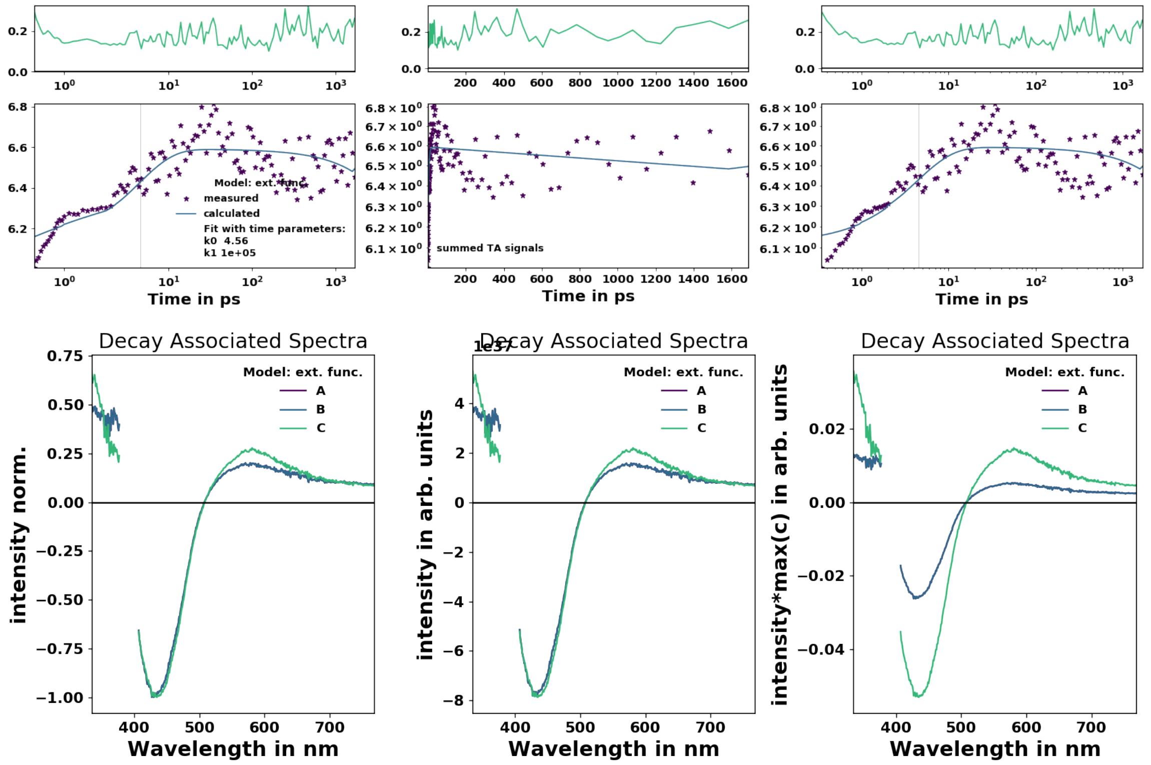

Figure S15: Fitted results: Kinetics of the sum of all wavelength (top) and the Decay Associated Spectra (bottom)

Save results

[14]:
```
savename = filename+'_own'
ta.Save_project(filename=savename,    # set save name
                path='results')    # set name of save folder

ta.Save_data(save_RAW=False,          # do not save pre-processed data
             save_Fit=True,           # save pre-processed and fitted data
             filename=savename,       # set save name
             path='results')       # set name of save folder
```

```
The project was saved to
(path)/Data/Fitting-2/results
```

4.3 Tutorial 03_KiMoPack_CompareFit.ipynb

[1]:
```
import os,sys
import pandas as pd
import numpy as np
import matplotlib,lmfit
import matplotlib.pyplot as plt
try:
    import KiMoPack.plot_func as pf
except:
    print("General installation did not work, import from the same folder as a␣
 ↪workaround")
    import plot_func as pf
```

```
Plot_func version 6.20
was imported from path:
 (path)
The current working folder is:
 (path)
```

4.3.1 Saving and Loading of Projects

In this example it is demonstrated how to work with saved *hdf5* projects. Upon saving a project all parameters of the analysis are dumped to the *hdf5* file. This includes the standard corrected (background, arrival time, scattercut, . . .) TA data as well as the fit results and parameter.

Herein, the TA projects of **Ru-dppz** collected in three solvents, namely dichloromethane (DCM), acetonitrile (ACN) and water (H_2O) at 400 nm excitation are loaded and compared. Firstly, the master project (here in ACN) is loaded to the `ta_ACN` object. Secondly all *hdf5* projects from a specified folder (including the data in DCM and H_2O) are loaded into the object `compare_projects`. For loading the comparison projects the function `GUI_open` is employed. You can either read all *hdf5* projects from a folder (`project_list='all'`) or you can select single projects from the file explorer (`project_list='gui'`).

[3]:
```
# initialize ta_ACN object including the TA data in ACN
ta_ACN = pf.TA(filename='TA_Ru-dppz_400nm_ACN_paral.hdf5',
               path=os.path.join('Data', 'Compare', 'Master'))
# initialize an object including the TA data in DCM and H2O
compare_projects=pf.GUI_open(project_list='all',
                             path=os.path.join('Data', 'Compare'))
# plot TA spectra of the master project
ta_ACN.Plot_fit_output(title='Master project, ACN, 400 nm', plotting=[3])
```

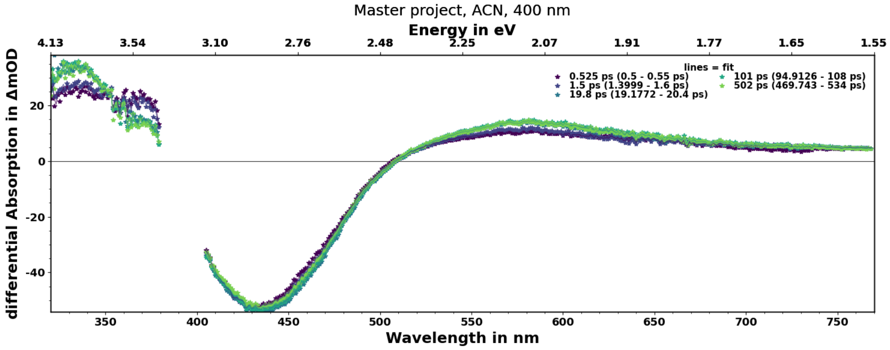

Figure S16: Initially fitted spectra of the master project

Upon loading the TA data of several projects, the user can compare the transient spectra by plotting multiple spectra into the same figure at given delay times (`rel_time`). Herein, the TA spectra of **Ru-dppz** obtained in different solvents, namely dichloromethan (DCM), acetonitrile (ACN) and water (H_2O) are compared.

4.3.1.1 General settings

- For a better comparison the data of each project can be normalized to the master object (in this example `ta_ACN`). For the normalization a normalization range is defined by the lower and upper limits of deay times and probe wavelengths (*e.g.* `norm_window=[0.5,0.7,420,470]`).
- In order to be able to compare the individual data sets well with each other, the use of a highly diverging colormap is recommended. Herein the colormap *Accent* is used. For more available maps see: https://matplotlib.org/stable/tutorials/colors/colormaps.html

[4]:
```
norm_window = [0.5,0.7,420,470]   # norm window
ta_ACN.cmap=pf.cm.Accent          # use a diverge colormap for comparison
```

4.3.1.2 Compare transient spectra

[5]:
```python
for delay_time in [1,10,100]:          #plot spectra for each selected delay time
    ta_ACN.Compare_at_time(fitted=False,   #compare pre-processed data
                 other=compare_projects,     #list of projects to compare
                 rel_time=[delay_time],      #selected decay times to compare
                 norm_window=norm_window)    #set norm window
```

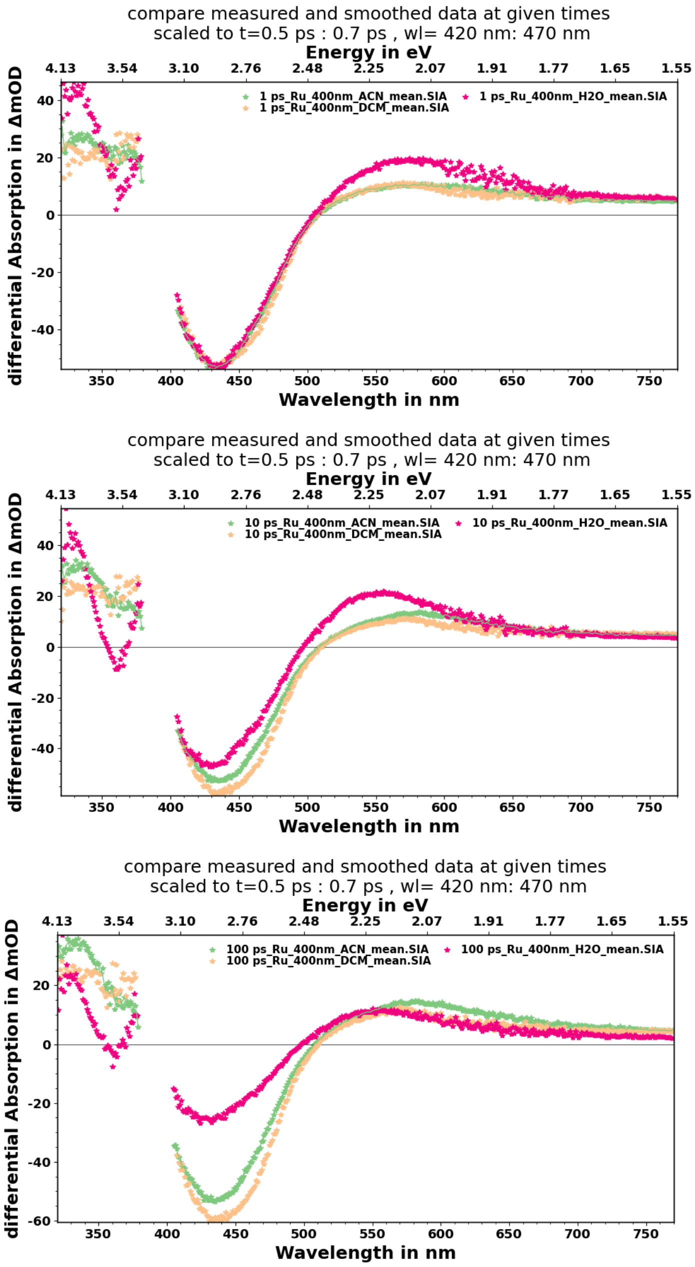

Figure S17: Comparing transient spectra at three different timepoints. 1ps top, 10ps middle and 100ps bottom. All spectra are normalized in the same window, allowing the direct comparision of the intensities.

4.3.1.3 Compare transient and external spectra

To compare the transient spectra at a certain delay time to an external spectrum (*e.g.* spectro-electro-chemistry or steady state absorption data), the user can pass a dataframe with such spectra to the `Compare_at_time`function. Herein, the absorption data of electrochemically unmodified **Ru-dppz** and its singly reduced form are loaded into a Pandas DataFrame with the wavelength column as index. In the example the scaled difference spectrum of the reduced and unreduced complex is plotted for comparison to the TA spectra of **Ru-dppz** in ACN and H_2O obtained at a delay time of 1 ps.

[5]:
```
# define name of external spectra
spectra_name = 'UVvis_SEC_Rudppz_ACN.dat'

# define path of external spectra
spectra_path = os.path.join(os.getcwd(), 'Data', 'Compare')

# create dataframe of external spectra
SEC_df = pd.read_csv(os.path.join(spectra_path, spectra_name), index_col=0,
                     sep="\t", header=0)
diff_spectrum=(SEC_df['red']-SEC_df['ocp'])*0.05   # create difference spectrum
diff_spectrum.name='Rudppz_ACN - difference'       # give it a name for the plot

ta_ACN.Compare_at_time(fitted=False,
                       rel_time=1.0,                 # selected delay time
                       other=compare_projects,       # list of projects to compare
                       spectra=diff_spectrum,        # external spectra to compare
                       norm_window=norm_window)      # set norm window
```

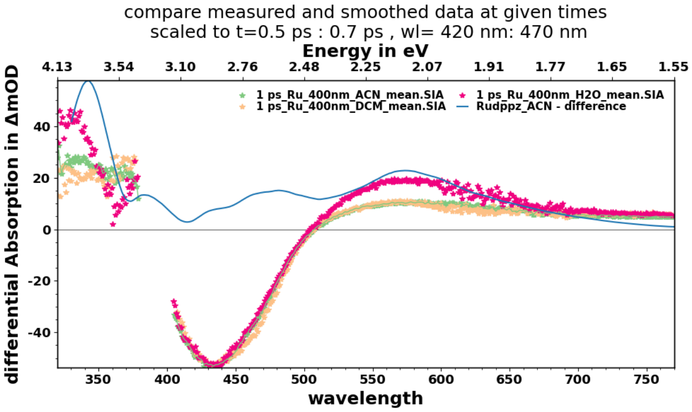

Figure S18: Spectra at 1 ps compared to external spectrum

4.3.1.4 Compare kinetic traces

The kinetic traces of several projects at a given probe wavelength (`rel_wave`) can be plotted into the same figure by the `Comapre_at_wave`function. This allows to compare the kinetics at various conditions (*e.g.* pump intensity, quencher concentration, solvent). A normalization window can be given at which all the plotted curves are normalized to. This window does not have to be in the plotted region. In this example the TA kinetics of the excited state absorption at 340 and 580 nm and the ground state bleach minimum at 440 nm in DCM, ACN and H_2O are compared.

[6]:
```
plt.close('all')
%matplotlib inline
%matplotlib qt
ta_ACN.cmap=pf.cm.Accent
for nm in [340,440,580]:   # plot kinetics at each selected wavelengths
    ta_ACN.timelimits=[-0.5,1500]                     # set timelimits of the plot
    ta_ACN.Compare_at_wave(fitted=False,              # plot preprocessed data
                           other=compare_projects,  # list of projects to compare
                           rel_wave=nm,           # selected wavelengths to compare
                           norm_window=norm_window) # norm window
```

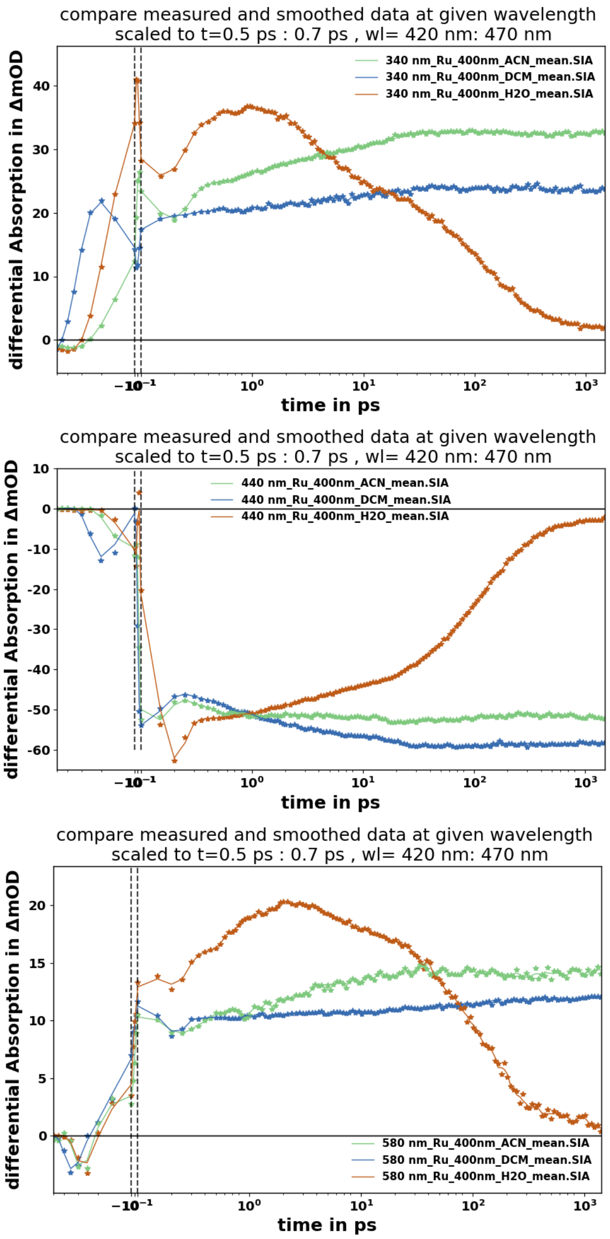

Figure S19: Comparing the Kinetics at three separate wavelength for the ruthenium complex in three different solvents. Note that all spectra were normalized to the same window. That allows the comparision of e.g. the residual excitation.

4.4 Tutorial `04_KiMoPack_ScanHandling.ipynb`

[1]:
```
import os,sys
import pandas as pd
import numpy as np
import matplotlib,lmfit
import matplotlib.pyplot as plt
try:
    import KiMoPack.plot_func as pf
except:
    print("General installation did not work, try to import from the same folder as␣
 ↪a workaround")
    import plot_func as pf
#qt is mandatory for the functioning of this module
%matplotlib qt
```

```
Plot_func version 6.2.9
was imported from path:  (path to local module)
The current working folder is:  (path to  current working folder)
```

Read and average single scans: Load single scans of a data set and select certain scans that are excluded from the summary (`Summarize_scans`). Therefore, up to two windows including lower and upper boundaries for delay times and probe wavelengths (*e.g.* `[1,10,500,700]`) can be defined. In that region the TA signals are integrated. The respective integrals of each scan are shown in an active plot. The scans to exclude from the average are selected by right-click on the respective data points in the active window. Advice: the GUI is sometimes hidden on the desktop. The window is recognizable with a little feather in the top left corner.

[ ]:
```
#simple usage, select all files with "ACN" in the name and "SIA" ending in the␣
 ↪folder with name "scans"
ta=pf.Summarize_scans('gui',                        # use gui to select files
                      list_to_dump='single',        # select single points to be removed
                      window1=[1,10,500,700])       # integration window
```

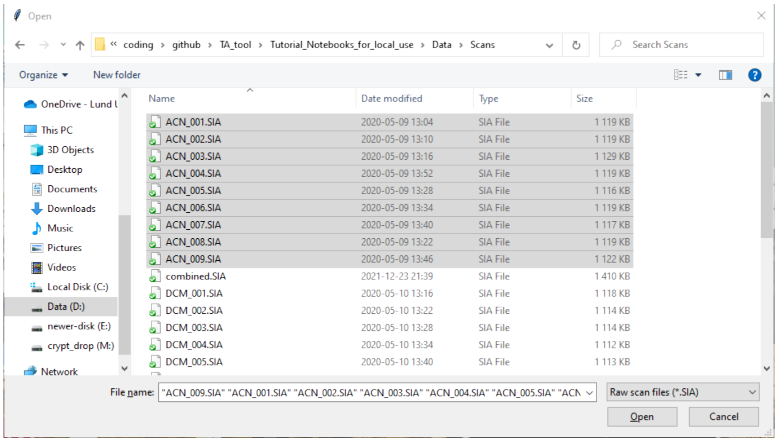

Figure S20: Gui for selecting the scans to be processed.

This is the advanced usage of the tool. By using "fileending", all files with a different ending are rejected. The "filename_part" works similar and looks if a certain string is in the filename, so can one e.g. filter all files with "ACN" in the name. The save_name is a useful option to keep track of the files. without it the file would be saved as "combined.SIA". The "list_of_scans" could be used to give e.g. a series of numbers that should be the last part of the filename. This is mainly useful if the option "return_list_of_names" is selected. Then e.g. multiple different selection series can be combined. See the Manual for more information on its usage.

```
[ ]: # Define a folder, This trick works on windows and linux
scanfolder = os.path.join(os.getcwd(), 'Data', 'Scans')
ta=pf.Summarize_scans(path_to_scans=scanfolder,    # define path of the scan files
                      list_of_scans=None,      # read all scans from the given folder
                      list_to_dump='single',     # select single points to be removed
                      window1=[1,10,500,700],    # integration window
                      window2=[1,10,410,470],    # integration window
                      fileending='.SIA',         # file extension, ignore rest
                      filename_part='ACN',       # part of the filenames to read
                      save_name='TA_Ru-dppz_400nm_'+'ACN'+'_mean.SIA')#set save name
```

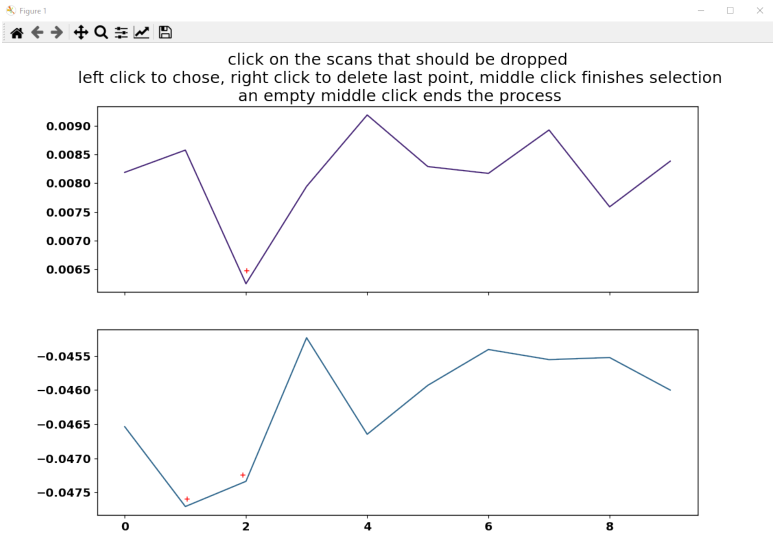

Figure S21: Gui that allows the selection of single scans to be dropped from the average.

References

[1] FEES, Joerg ; KAIM, Wolfgang ; MOSCHEROSCH, Michael ; MATHEIS, Walter ; KLIMA, Jiri ; KREJCIK, Michael ; ZALIS, Stanislav: Electronic structure of the "molecular light switch" bis(bipyridine)dipyrido[3,2-a:2',3'-c]phenazineruthenium(2+). Cyclic voltammetric, UV/visible and EPR/ENDOR study of multiply reduced complexes and ligands. in: *Inorganic Chemistry* 32 (1993), jan, nr. 2, 166–174. http://dx.doi.org/10.1021/ic00054a009. – DOI 10.1021/ic00054a009. – ISSN 0020–1669

[2] FEES, Jörg ; KETTERLE, Michael ; KLEIN, Axel ; FIEDLER, Jan ; KAIM, Wolfgang: Electrochemical, spectroscopic and EPR study of transition metal complexes of dipyrido[3,2-a:2',3'-c]phenazine. in: *Journal of the Chemical Society, Dalton Transactions* (1999), nr. 15, 2595–2600. http://dx.doi.org/10.1039/a903417j. – DOI 10.1039/a903417j. – ISSN 03009246

[3] BRENNAMAN, Matthew K. ; MEYER, Thomas J. ; PAPANIKOLAS, John M.: [Ru(bpy) 2 dppz] 2+ Light-Switch Mechanism in Protic Solvents as Studied through Temperature-Dependent Lifetime Measurements. in: *The Journal of Physical Chemistry A* 108 (2004), nov, nr. 45, 9938–9944. http://dx.doi.org/10.1021/jp0479670. – DOI 10.1021/jp0479670. – ISSN 1089–5639

[4] BRENNAMAN, Matthew K. ; ALSTRUM-ACEVEDO, James H. ; FLEMING, Cavan N. ; JANG, Paul ; MEYER, Thomas J. ; PAPANIKOLAS, John M.: Turning the [Ru(bpy) 2 dppz] 2+ Light-Switch On and Off with Temperature. in: *Journal of the American Chemical Society* 124 (2002), dec, nr. 50, 15094–15098. http://dx.doi.org/10.1021/ja0279139. – DOI 10.1021/ja0279139. – ISSN 0002–7863

[5] OLSON, E. J. C. ; HU, D. ; HÖRMANN, A. ; JONKMAN, A. M. ; ARKIN, M. R. ; STEMP, E. D. A. ; BARTON, J. K. ; BARBARA, P. F.: First Observation of the Key Intermediate in the "Light-Switch" Mechanism of [Ru(phen) 2 dppz] 2+. in: *Journal of the American Chemical Society* 119 (1997), nov, nr. 47, 11458–11467. http://dx.doi.org/10.1021/ja971151d. – DOI 10.1021/ja971151d. – ISSN 0002–7863

[6] OLOFSSON, Johan ; ÖNFELT, Björn ; LINCOLN, Per: Three-State Light Switch of [Ru(phen) 2 dppz] 2+ : Distinct Excited-State Species with Two, One, or No Hydrogen Bonds from Solvent. in: *The Journal of Physical Chemistry A* 108 (2004), may, nr. 20, 4391–4398. http://dx.doi.org/10.1021/jp037967k. – DOI 10.1021/jp037967k. – ISSN 1089–5639

[7] KUHNT, Christian ; KARNAHL, Michael ; TSCHIERLEI, Stefanie ; GRIEBENOW, Kristin ; SCHMITT, Michael ; SCHÄFER, Bernhard ; KRIECK, Sven ; GÖRLS, Helmar ; RAU, Sven ; DIETZEK, Benjamin ; POPP, Jürgen: Substitution-controlled ultrafast excited-state processes in Ru-dppz-derivatives. in: *Physical Chemistry Chemical Physics* 12 (2010), nr. 6, 1357–1368. http://dx.doi.org/10.1039/B915770K. – DOI 10.1039/B915770K. – ISSN 1463–9076

[8] POURTOIS, Geoffrey ; BELJONNE, David ; MOUCHERON, Cécile ; SCHUMM, Stephan ; KIRSCH-DE MESMAEKER, Andrée ; LAZZARONI, Roberto ; BRÉDAS, Jean-Luc: Photophysical Properties of Ruthenium(II) Polyazaaromatic Compounds: A Theoretical Insight. in: *Journal of the American Chemical Society* 126 (2004), jan, nr. 2, 683–692. http://dx.doi.org/10.1021/ja034444h. – DOI 10.1021/ja034444h. – ISSN 0002–7863

[9] OLOFSSON, Johan ; WILHELMSSON, L. M. ; LINCOLN, Per: Effects of Methyl Substitution on Radiative and Solvent Quenching Rate Constants of [Ru(phen) 2 dppz] 2+ in Polyol Solvents and Bound to DNA. in: *Journal of the American Chemical Society* 126 (2004), dec, nr. 47, 15458–15465. http://dx.doi.org/10.1021/ja047166a. – DOI 10.1021/ja047166a. – ISSN 0002–7863

[10] Önfelt, Björn ; Olofsson, Johan ; Lincoln, Per ; Nordén, Bengt: Picosecond and Steady-State Emission of [Ru(phen) 2 dppz] 2+ in Glycerol: Anomalous Temperature Dependence. in: *The Journal of Physical Chemistry A* 107 (2003), feb, nr. 7, 1000–1009. http://dx.doi.org/10.1021/jp0269266. – DOI 10.1021/jp0269266. – ISSN 1089–5639

[11] Damrauer, Niels H. ; Cerullo, Giulio ; Yeh, Alvin ; Boussie, Thomas R. ; Shank, Charles V. ; McCusker, James K.: Femtosecond Dynamics of Excited-State Evolution in [Ru(bpy)3]2+. in: *Science* 275 (1997), jan, nr. 5296, 54–57. http://dx.doi.org/10.1126/science.275.5296.54. – DOI 10.1126/science.275.5296.54. – ISSN 00368075

[12] Stark, Charles W. ; Schreier, Wolfgang J. ; Lucon, Janice ; Edwards, Ethan ; Douglas, Trevor ; Kohler, Bern: Interligand Electron Transfer in Heteroleptic Ruthenium(II) Complexes Occurs on Multiple Time Scales. in: *The Journal of Physical Chemistry A* 119 (2015), may, nr. 20, 4813–4824. http://dx.doi.org/10.1021/acs.jpca.5b01770. – DOI 10.1021/acs.jpca.5b01770. – ISSN 1089–5639

[13] Wallin, Staffan ; Davidsson, Jan ; Modin, Judit ; Hammarström, Leif: Femtosecond Transient Absorption Anisotropy Study on [Ru(bpy) 3] 2+ and [Ru(bpy)(py) 4] 2+ . Ultrafast Interligand Randomization of the MLCT State. in: *The Journal of Physical Chemistry A* 109 (2005), jun, nr. 21, 4697–4704. http://dx.doi.org/10.1021/jp0509212. – DOI 10.1021/jp0509212. – ISSN 1089–5639

[14] Juris, A. ; Balzani, V. ; Barigelletti, F. ; Campagna, S. ; Belser, P. ; Zelewsky, A. von: Ru(II) polypyridine complexes: photophysics, photochemistry, eletrochemistry, and chemiluminescence. in: *Coordination Chemistry Reviews* 84 (1988), mar, 85–277. http://dx.doi.org/10.1016/0010-8545(88)80032-8. – DOI 10.1016/0010–8545(88)80032–8. – ISSN 00108545

[15] Uhlig, Jens: *KiMoPack Anaconda installation (using conda package manager) version 6.6.2*. https://conda.anaconda.org/erdzeichen. Version: 2022

[16] Uhlig, Jens: *KiMoPack PyPi installation (using pip package manager) version 6.6.2*. https://pypi.org/project/KiMoPack/. Version: 2022
