# Supplementary figures and images for: KiMoPack: A python Package for Kinetic Modeling of the Chemical Mechanism
Source: J Phys Chem A. 2022 Jun 14;126(25):4087–99. doi: 10.1021/acs.jpca.2c00907 (PMC9251768; doi:10.1021/acs.jpca.2c00907)

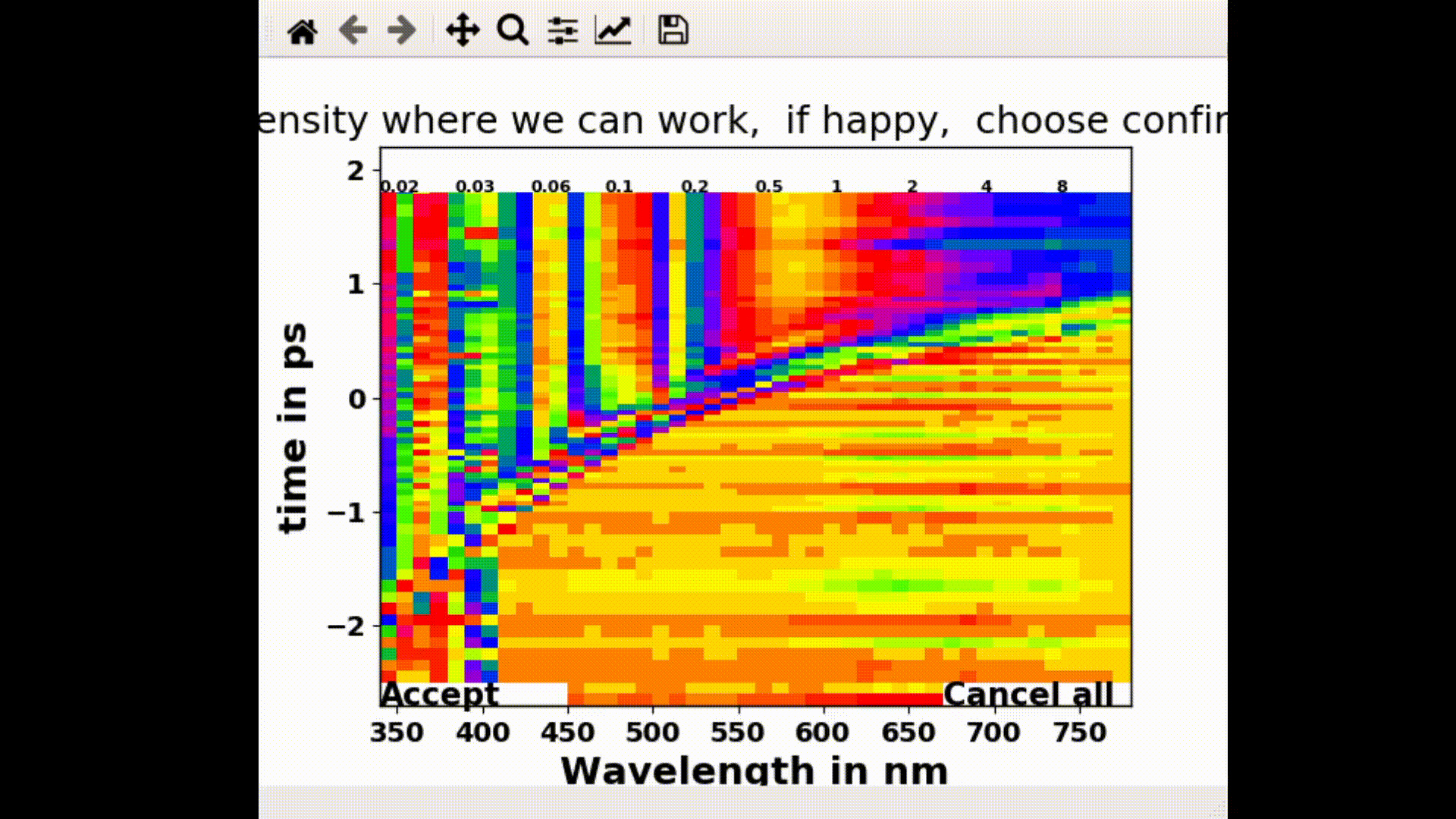

Supplement: Supplementary file 2 — jp2c00907_si_002.zip [file jp2c00907_si_002.zip › img/Cor_Chirp.gif]

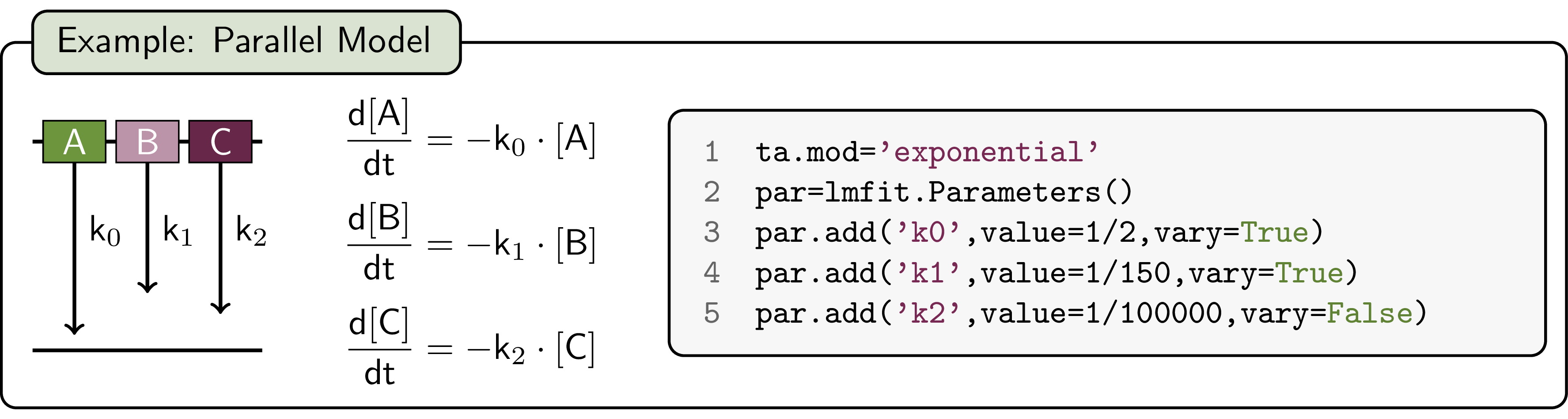

Supplement: Supplementary file 2 — jp2c00907_si_002.zip [file jp2c00907_si_002.zip › img/Fig1_parallel_model.png]

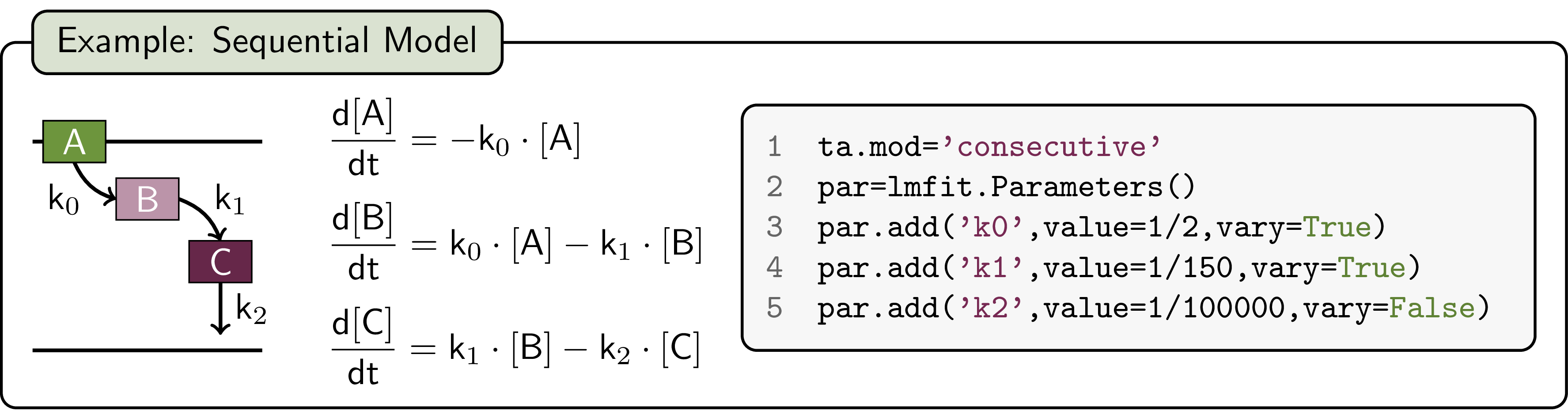

Supplement: Supplementary file 2 — jp2c00907_si_002.zip [file jp2c00907_si_002.zip › img/Fig2_consecutive_model.png]

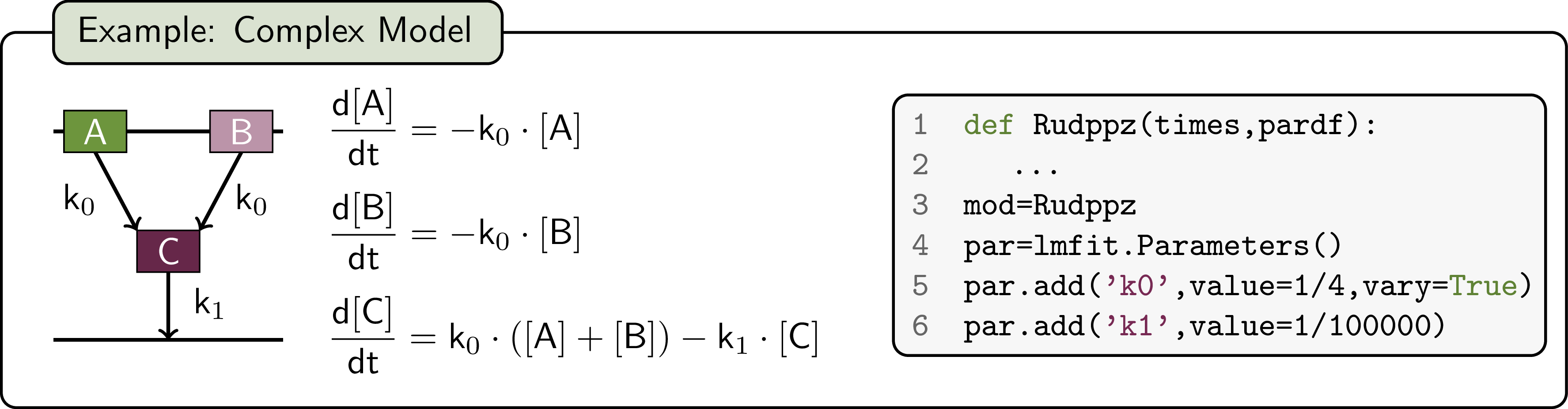

Supplement: Supplementary file 2 — jp2c00907_si_002.zip [file jp2c00907_si_002.zip › img/Fig3_complex_model.png]
